# Identification of risk factors for patients with diabetes: diabetic polyneuropathy case study

**DOI:** 10.1186/s12911-020-01215-w

**Published:** 2020-08-24

**Authors:** Oleg Metsker, Kirill Magoev, Alexey Yakovlev, Stanislav Yanishevskiy, Georgy Kopanitsa, Sergey Kovalchuk, Valeria V. Krzhizhanovskaya

**Affiliations:** 1 Almazov National Medical Research Centre, Saint-Petersburg, Russia; 2 ITMO University, Birzhevaya 4, Saint Petersburg, Russia; 3 University of Amsterdam, Amsterdam, The Netherlands

**Keywords:** Polyneuropathy, Machine learning, Risk factors, Clinical decision support

## Abstract

**Background:**

Methods of data mining and analytics can be efficiently applied in medicine to develop models that use patient-specific data to predict the development of diabetic polyneuropathy. However, there is room for improvement in the accuracy of predictive models. Existing studies of diabetes polyneuropathy considered a limited number of predictors in one study to enable a comparison of efficiency of different machine learning methods with different predictors to find the most efficient one. The purpose of this study is the implementation of machine learning methods for identifying the risk of diabetes polyneuropathy based on structured electronic medical records collected in databases of medical information systems.

**Methods:**

For the purposes of our study, we developed a structured procedure for predictive modelling, which includes data extraction and preprocessing, model adjustment and performance assessment, selection of the best models and interpretation of results. The dataset contained a total number of 238,590 laboratory records. Each record 27 laboratory tests, age, gender and presence of retinopathy or nephropathy). The records included information about 5846 patients with diabetes. Diagnosis served as a source of information about the target class values for classification.

**Results:**

It was discovered that inclusion of two expressions, namely “nephropathy” and “retinopathy” allows to increase the performance, achieving up to 79.82% precision, 81.52% recall, 80.64% F1 score, 82.61% accuracy, and 89.88% AUC using the neural network classifier. Additionally, different models showed different results in terms of interpretation significance: random forest confirmed that the most important risk factor for polyneuropathy is the increased neutrophil level, meaning the presence of inflammation in the body. Linear models showed linear dependencies of the presence of polyneuropathy on blood glucose levels, which is confirmed by the clinical interpretation of the importance of blood glucose control.

**Conclusion:**

Depending on whether one needs to identify pathophysiological mechanisms for one’s prospective study or identify early or late predictors, the choice of model will vary. In comparison with the previous studies, our research makes a comprehensive comparison of different decisions using a large and well-structured dataset applied to different decision support tasks.

## Background

Every third patient with diabetes suffers from diabetic polyneuropathy (DPN) [1]. This complication is a severe problem for a chronic patient because it can be accompanied by neuropathic pain and lead to a decrease in the quality of life. Neuropathic pain significantly disrupts sleep, negatively affects daily activity and life satisfaction. Due to the asymptomatic nature, it is important to have methods for identifying and predicting the development of this complication [2]. Diabetes is usually accompanied by other comorbid conditions such as hypertension, chronic heart failure and others that should be considered when analyzing risk factors [3].

The earlier diagnostics results in efficient management of the disease. Machine learning methods can support doctors with a preliminary judgment about diabetic polyneuropathy based on routinely collected physical examination data, and can support doctors in their decision-making process.

Selecting the valid features and the correct classifier are the most important problems for machine learning methods [4, 5]. Predictive models for diabetes already provide highly accurate prognosis of the disease.

Several studies on the prediction of diabetes mellitus that utilize Linear regression (LR) [6, 7] developed predictive models based on the records with up to 967 independent variables available from large electronic health record archives that resulted in up to 80% Area Under The Curve Receiver Operating Characteristics (AUC-ROC) [6] and enabled the formulation the most likely trajectory of comorbidities [7].

Application of the ensemble models approach based on the naïve Bayes, Linear regression (LR), Instance-Based Learner, support vector machine (SVM) [8], artificial neural networks (ANN) [9], decision trees [10], and random forests (RF) help to increase the accuracy of diabetes prediction up to 74% [11, 12].

Prediction of risks of specific complications of diabetes can achieve higher accuracy than general diabetes prediction models. For example, Sudharsan et al. [13] applied methods of machine learning to predict hypoglycemia in patients with diabetes as a result of insulin treatment. The authors used a variable number of blood glucose measurements to predict hypoglycemia (low blood glucose). According to the research, the RF model showed the best results at 10 measurements per week (92% sensitivity and 70% specificity). When using additional data on prescribed medications and reducing the prognosis window to 1 h, the specificity of the model increases to 90%.

Specific predictive models for diabetes polyneuropathy are based on screening methods, for example Nerve conduction studies (NCS) [14] can reach up to AUC 65.8–84.7% for the conditional diagnosis of DPN in primary care.

Prediction methods that utilize data from personal health records deal with large non-specific datasets with different prediction methods. Li et al. [15] utilized 30 independent variables, which allowed to implement a model with AUC = 88.63% for a Multilayer perceptron (MLP). Linear regression (LR) based methods [6, 16] produced up to AUC = 80.0%.

Huang et al. [17] developed a DT-based model to predict diabetic nephropathy. Their method-based laboratory analyses and genetic data in conjunction with patient-field-based rules to achieve maximum prediction accuracy. Depending on the gender of a patient, the model uses a set of variables that show the highest results in the corresponding group. According to the study, the model achieved the accuracy of 78.50%, specificity of 80.64%, and sensitivity of 81.40%.

Lagani et al. [18, 19] developed models to determine the risk of diabetes-related complications. Complications considered by the authors include cardiovascular disease, hypoglycemia, ketoacidosis, microalbuminuria, proteinuria, neuropathy, and retinopathy. For all outcomes, the authors determined the smallest set of clinical parameters and the algorithm to achieve the highest accuracy of diagnosis. The set of developed models achieves the accuracy from 62 to 83% depending on the specific complication.

The progress of polyneuropathy depends not only on the duration of diabetes but also on the treatment. It is known that with adequate control of blood glucose level, the incidence of polyneuropathy after 15 years from the development of diabetes does not exceed 10%, and with poor control of glycemia increases to 50%. Also, no direct relationship was discovered between the severity of diabetes and the progression of polyneuropathy (for example, severe forms of polyneuropathy can be observed in patients with a relatively mild course of diabetes). According to EURODIAB IDDM Complication Study (2001), the risk factors for polyneuropathy were: old age, duration of diabetes mellitus, hemoglobin level, excessive weight, proliferative diabetic retinopathy, high level of low-density lipoproteins, cardiovascular disease. According to Seattle Prospective Diabetic Foot Study (1996), new factors were found, including increased diastolic blood pressure, ketoacidosis, increased triglyceride levels, and microalbuminuria. Currently, the theory of “metabolic memory” (2007) is being developed, according to which the first (early) metabolic or inflammatory changes due to certain inertia of the pathological process can have a long-term effect and contribute to the further progression of late complications of diabetes mellitus. In clinical practice, the importance of early diagnosis of polyneuropathy is often underestimated by many specialists, and many cases remain unrecognized. Previously, the prognostic significance of risk factors for the occurrence and progression of polyneuropathy was not assessed in the studies. Isolation of high-risk groups and the progress of polyneuropathy among patients with diabetes mellitus will allow to improve the quality of medical care and reduce the number of disabling forms of the disease. Prediction model can also be used to set-up focused clinical studies with the hypotheses supported by real world evidences (RWE).

Methods of data mining and analytics can be efficiently applied in medicine to develop models that use patient-specific data to predict the development of diabetic polyneuropathy, however there is still space to improve the efficiency of the predictive models. None of the studies dealing with diabetes polyneuropathy considered any significant number of predictors in one study to enable a comparison of efficiency of different machine learning methods with different predictors to find the most efficient one. The studies [11, 12, 18, 19] that dealt with applying machine learning to diabetes complications did not provide a comprehensive comparison of different decision support models using a well-structured and large enough dataset applied to different decision support tasks.

The purpose of this study is the implementation of machine learning methods for early identification of the risk of diabetes polyneuropathy based on structured electronic medical records collected in databases of medical information systems. The models developed in this study consider comorbidity with retinopathy and nephropathy.

## Methods

The data from the medical information system of Almazov specialized medical center was used as the empirical basis of the study. The unique conditions of combining several specialized departments into a single system create prerequisites for the validity of this study. The medical center has diagnostic, intensive care, rehabilitation, and other departments. This medical center is one of the most well-equipped in Russia. The degree of digitalization of treatment processes is also at a high level. On this basis, it is possible to extract data from the medical system with a sufficient level of compliance with the real process. The local ethics committee of ITMO university approved the study.

**Fig. 1:**
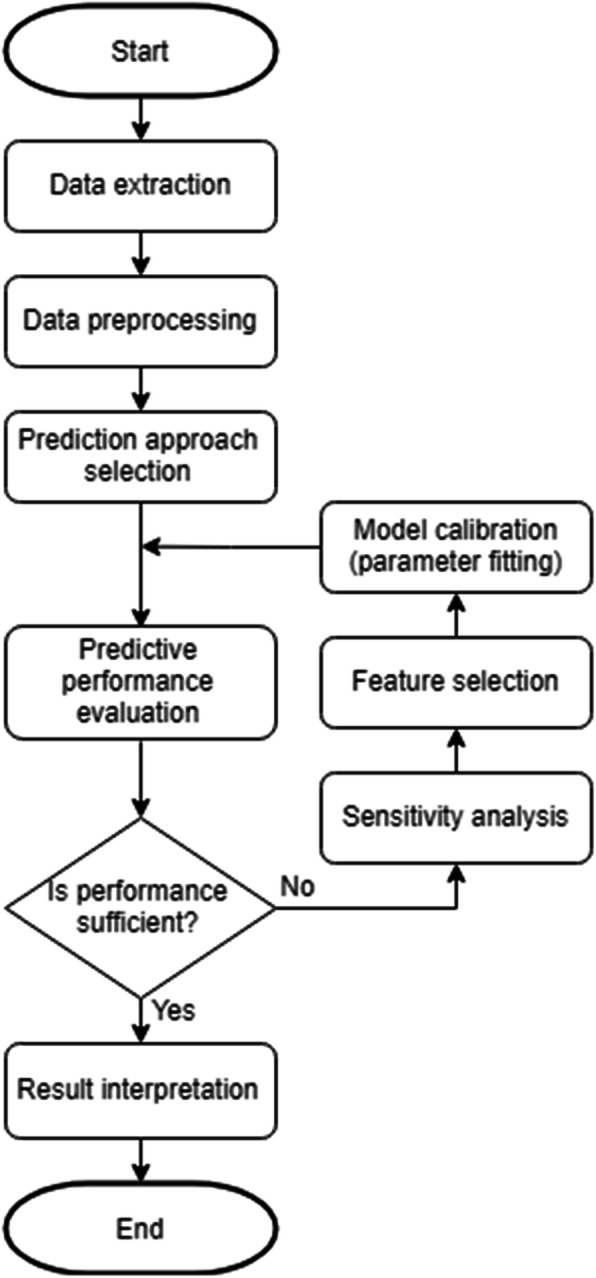
Workflow of predictive model development training

For our study, we’ve applied a basic procedure for predictive modelling (Fig. 1), which includes data extraction and preprocessing, models’ adjustment and performance assessment, selection of the best models, and interpretation of the results. Further in this section, basic methods and technologies are described. For this study we used Python 3.6.3 and scikit-learn 0.19.1 [20] as the basic framework for machine learning models.

### Data extraction and preprocessing

The dataset contains the total number of 238,590 laboratory records. Each record contains patient and episode identifiers, a timestamp, and a number of measured parameters, the total number of which is **31** (27 laboratory tests, retinopathy, nephropathy, age, and gender).

The patients were eligible for the study based on the following inclusion and exclusion criteria:

Inclusion criteria:

Age > = 16 years old.

Diagnoses:

- E08 Diabetes mellitus due to underlying condition
- E09 Drug or chemical induced diabetes mellitus
- E10 Type 1 diabetes mellitus
- E11 Type 2 diabetes mellitus

Timeframe: Cases were open form 03-07-2010 to 22-08-2017.

Exclusion criteria:

Age < 16 years old.

E12 Malnutrition-related diabetes mellitus.

O24 Diabetes mellitus in pregnancy, childbirth, and the puerperium.

Case opened outside of a timeframe 03-07-2010 to 22-08-2017.

The records cover the time period from 03 to 07-2010 to 22-08-2017 and include information about 5846 patients with diabetes. Diagnosis served as a source of information about the target class values for classification (whether or not a patient had developed polyneuropathy).

In this article we focus on the analysis of complication factors in patients in general. Diabetes mellitus is a chronic disease that a patient acquires for life we did not divide the dataset into episodes.

In the first phase of the data preprocessing, we selected a set of 31 parameters with the largest populations of patients and the longest average time series length and filtered out the rest, normalized the remaining values by parameter medians, and interpolated and extrapolated the patients’ data to convert it into regularly sampled time series having equal lengths.

Laboratory test datasets were extracted from the medical database to tables for validation and analysis. All data was anonymized. The laboratory tests included:

**Table:** 

1. Hemoglobin (HGB), 2. Leukocytes (LEU), 3. Platelets (PLT), 4. pH, 5. Mean platelet volume (MPW), 6. Creatinine, 7. Mean cell hemoglobin (MCH), 8. Neutrophils (NEUT), 9. Mean corpuscular volume (MCV), 10. Cholesterol, 11. Glucose, 12. Procalcitonin (PCT), 13. Red blood cell distribution width (RDW), 14. Alanine transaminase (ALT),	15. Bilirubin, 16. Platelet distribution width (PDW), 17. High-density lipoprotein (HDL), 18. Aspartate aminotransferase (AST), 19. White blood count (WBC), 20. Troponin, 21. Monocytes, 22. Bilirubin, 23. Red blood cell count (RBC), 24. Triglycerides, 25. Hematocrit (HCT), 26. Low-density lipoproteins (LDL), 27. Blood in urine (BLD).

In addition to the laboratory data, the dataset contained patients’ gender, age, and the presence of comorbidities: retinopathy, nephropathy (true or false).

We removed the patients with the insufficient amount of data (< 6 parameters) and no data for the retinopathy and nephropathy from the dataset. We also removed 1% of values having the highest z-score to filter out some obvious outliers.

We employed three approaches to preprocessing of the time series information:

1. Replacement of each time series with its last value;
2. Replacement of each time series with three of its characteristics: mean, length, and standard deviation;
3. Replacement of each time series with its maximum, with the exception of hemoglobin time series, which were replaced with their minimums.

After that, we applied two strategies of dealing with missing values to ensure that all patients have the same set of variables:

1. Replacement of missing data with the medians of the corresponding parameters;
2. Deletion of parameters that have too many missing values (> 500 by default) and removal of all patients that have any missing values in the remaining parameters.

The resulting dataset was randomly split into train (80%) and test (20%) samples. All the models were implemented using a train data set.

### Cluster identification in diabetes population

To identify subclasses within the class of patients with diabetes, the clustering problem was solved. The scikit-learn library’s method of T-distributed Stochastic Neighbour Embedding (T-SNE) was used. T-SNE was used as the most efficient method to organize unsupervised learning. It excludes bias related to the known number of clusters (e.g. this bias exists in the principal component analysis). In comparison with SNE, T-SNE uses a Student distribution that minimizes the influence of outliers that are common in medical data sets [21, 22]. The values presented in Table 1 for T-SNE parameters in scikit-learn were selected after several iterations with expert interpretation.

**Table 1:** T-SNE parameters

Parameter	Value	Parameter	Value
perplexity	30.0	metric	Euclidean
early_exaggeration	12.0	init	random
learning_rate	200.0	verbose	0
n_iter	1000	random_state	None
n_iter_without_progress	300	method	barnes_hut
min_grad_norm	1e-07	angle	0.5

All the parameters of the dataset were used for training the clustering model. The features were selected on the basis of correlation analysis for the target class. Grid search was used to select the optimal values of hyperparameters (Table 1).

### Sensitivity analysis and grid search for the classification model

Each experiment ran in the setting of stratified 5-fold cross-validation i.e., random 80% of training dataset was used for training and random 20% of training dataset for testing. Target class ratios in the folds were preserved. For the performance assessment of SVM and DT classifiers, we ran it 100 times; 100 × 5-fold cross-validation resulted in 500 predictions. All the measurements were performed separately per dataset and per model parameter value to determine the best parameters for classifiers as well as optimal data preprocessing. After determining the optimal dataset and model parameters, we performed a validation with the testing dataset. As an additional performance assessment score, we used AUC of ROC, which represents the trade-off between sensitivity and specificity of the model. We used a series of classification models available within scikit-learn as a pool for the selection of the best predictive methods to be applied within the proposed scheme. A summary of the required models parameters is presented in Table 2.

**Table 2:** Classification models

Model	Parameter	Value
Artificial Neural Network (ANN) (Multilayer Perceptron)	Number of nodes in the hidden layer	From 5 to 20 features
Support Vector Machine (SVM)	Kernel function	Linear
Decision tree	Max tree depth	2, 4, 8, 16
Linear regression	Normalize; n_jobs	False; none
Logistic regression	Criterion; Min_samples_split	Gini; 2

### Model evaluation

All the models were evaluated using a test data set. To evaluate the results of classification, we used common measures for machine learning models: accuracy, precision, recall, and F-measure. In the case of medical data, it is more expedient to use the recall metric, since the most critical problem of predicting diagnoses is the error of the first kind (the disease exists, but is not classified).

We performed all the experiments with all the classifiers on the full dataset (with comorbidities) and reduced dataset (without comorbidities) to measure the influence of comorbidities on the resulting performance of the classifiers.

### Results interpretation

For the interpretation of the results, we employed LIME [23], an explanation technique that explains the predictions of each classifier. We applied it to all classifiers we experimented with except the linear regression classifier, since it already has the exact solution for what the method is trying to approximate. Every classifier was trained 5 times using randomly drawn 80% of all the patients, then the predicted outcomes for the remaining 20% of patients were produced, after which each prediction was interpreted by LIME one-by-one. For every prediction, LIME produced a set of linear model coefficients corresponding to the variables, which can be interpreted as a set of variable contributions (given that the input was normalized).

The interpretation was carried out by two independent experts in the field of vascular disease and neurology, as well as in the field of endocrine diseases. The interpretation method included validation of the results and exclusion of invalid values. Further, by cross-comparison, conflicting elements were excluded if at least one expert considered it contradictory. After that, the conclusions of both experts were combined.

## Results

### Data preprocessing

After the patients with insufficient amount of data were removed from the dataset, it contained 5425 patients, 2342 (43.17%) of which did and 3083 (56.83%) did not develop polyneuropathy. Applying three ways to preprocess the time series and two approaches of handling missing data resulted in six datasets to apply our methods to. The summary of these datasets is presented in Table 3.

**Table 3:** Properties of datasets after preprocessing

	Time series replacement		
	Last values	Stats	Maximums/Minimums
Fill in the missing data	5425 patients, 31 parameters, 43.17% developed polyneuropathy	5425 patients, 31 parameters, 43.17% developed polyneuropathy	5425 patients, 31 parameters, 43.17% developed polyneuropathy
Filter out the missing data	5094 patients, 19 parameters, 43.62% developed polyneuropathy	5094 patients, 31 parameters, 43.62% developed polyneuropathy	5094 patients, 19 parameters, 43.62% developed polyneuropathy

### Subclasses in diabetes population

**Fig. 2:**
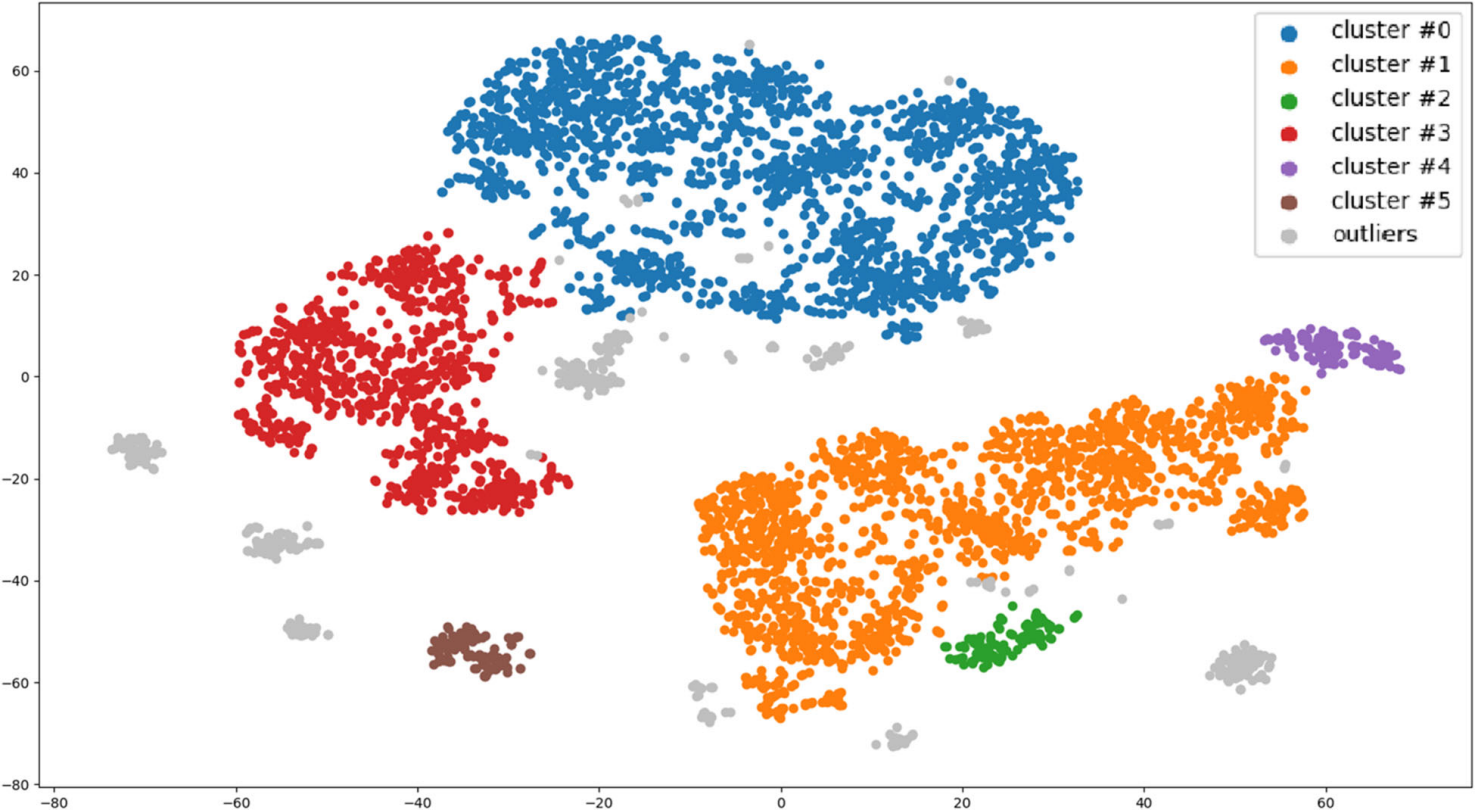
Clustering patients with diabetes using T-SNE machine learning method

As a result of clustering using T-SNE, 6 subclasses were identified (Fig. 2, Table 4).

**Table 4:** Cluster description

Cluster №	Count	Polyneuropahy, %	Age, years median	Age, years STD	Males, %	Females, %
Cluster #0	1877	59	65	9.79	0	100
Cluster #1	1628	43	60	13.74	100	0
Cluster #2	101	40	61	9.14	100	0
Cluster #3	930	0	31	6.53	0	100
Cluster #4	119	0	21	6.10	100	0
Cluster #5	111	23	39	20.29	99	1

There are two large clusters (Cluster #0 and Cluster #3) that are opposite in the percentage of patients with polyneuropathy, namely the smallest and the largest rate of polyneuropathy. Cluster #0 has an increased rate polyneuropathy: 59%. In cluster #3, on the contrary, the reduced number of patients with polyneuropathy is 0%. Both clusters are female. In male clusters #1 and #2, the percentage is close to the average of 40%. Cluster #4 is slightly below average. Cluster #5 is characterized by low polyneuropathy rates. When considering age distribution in the clusters, in most cases polyneuropathy can occur in patients that are 45 years or older. Polyneuropathy was observed only in clusters #0, 1, 2, and 5. No polyneuropathy was observed in clusters 3 and 4. This conclusion supports the validity of the results as they correlate with clinical practices. In clusters 0, 1, and 2, patients are about the same age. The peculiarity of cluster 1 is that there is an outlier on the left and it is male in contrast to cluster 0. Cluster 3 patients are young men younger than patients in clusters 0, 1, and 2 and also male. Cluster 5 patients of different ages with a small rate of polyneuropathy are mostly men. It is evident that female gender and age of more than 50 years is a risk factor for the development of polyneuropathy in patients with diabetes mellitus.

### Correlation analysis and feature importance

**Fig. 3:**
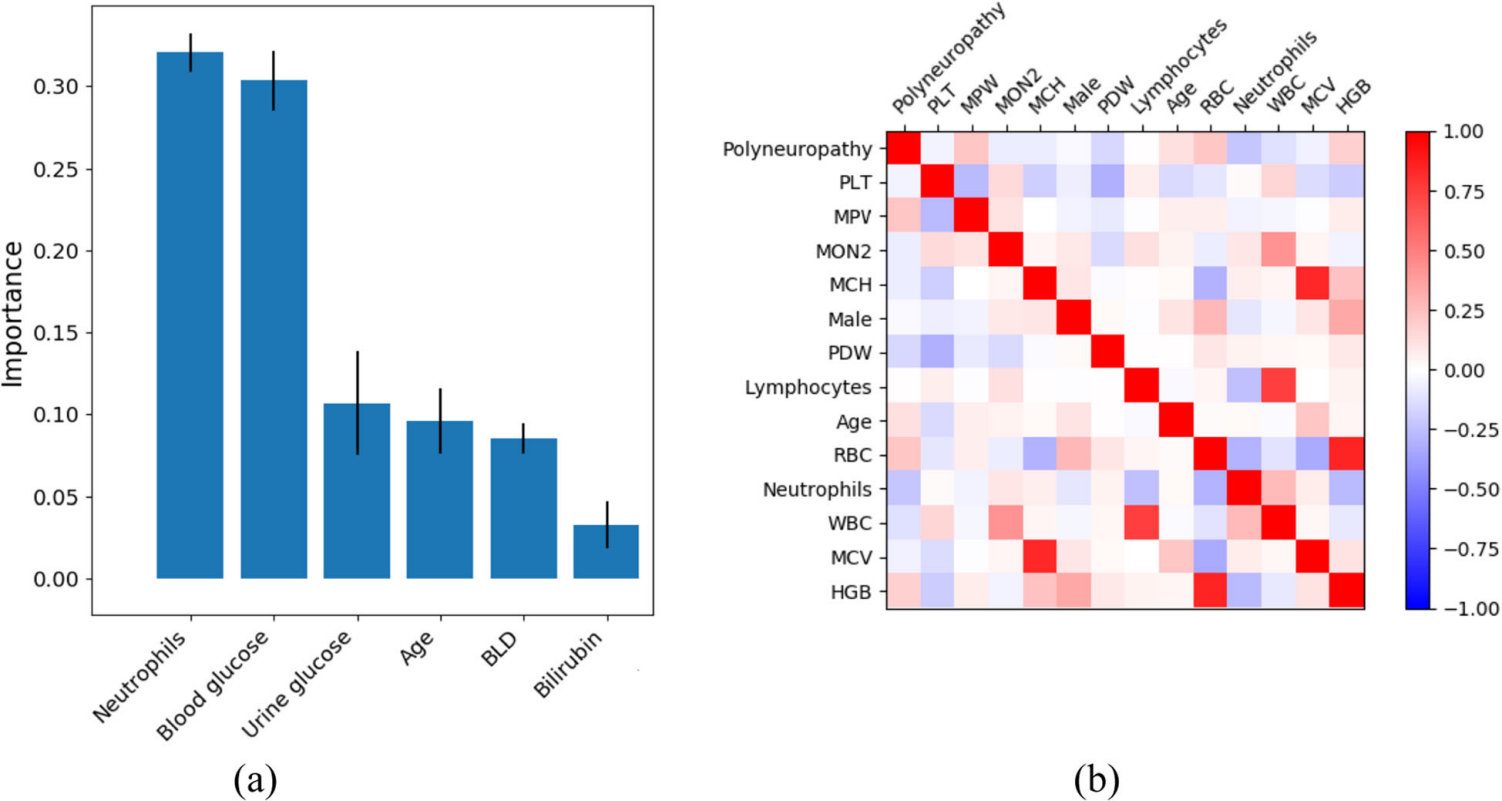
The feature importance of decision tree (**a**). The correlation matrix of features and complication (**b**)

Figure 3a shows the feature importance of the decision tree trained further. Figure 3b shows an example of a correlation matrix for selected parameters of the patient and polyneuropathy.

The most significant features are the levels of neutrophils and glucose level in blood and urine. The correlation matrix shows the high correlation of MPV, RBC, and HGB with polyneuropathy.

### Results of sensitivity analysis and grid search for the classification model

This section presents the results of sensitive analysis and model identification. The selected predictive models were trained and compared according to the methodology described earlier in Section 2. The key performance indicators for the validation of the best selected options are presented in Table 5. Further subsections provide more details for each of the models.

**Table 5:** Performance of the classifiers without comorbidities

Model	Precision	Recall	F1 score	Accuracy
ANN	0.6736	**0.8090**	0.7342	0.7471
SVM	0.6817	0.7655	0.7210	0.7443
Decision tree	0.6526	0.7302	0.6865	0.7039
Linear Regression	0.6777	0.7911	**0.7299**	**0.7472**
Logistic Regression	**0.6826**	0.7693	0.7232	0.7384

#### Results of artificial neural network classifier and sensitivity analysis

As it was stated before, sensitivity analysis for ANN consists of defining the optimal hidden layer configuration. It was decided to experiment only with single-hidden-layer networks, since it is commonly considered sufficient for simple machine learning tasks, which was confirmed by a number of experiments. For each of our datasets, we run 10 × 5-fold validation for varying number of nodes in the hidden layer of the network to determine the optimal value for each preprocessing and filtering option. Figures A3.1–A3.6 in Additional file 3 present the results of the process.

According to the figures, the method’s performance is growing as the number of nodes in the hidden layer increases up to the certain critical value after which performance remains relatively stable. The resulting curves are presented in Figures A3.5–A3.12 in Additional file 3.

#### Results of SVM

As it was mentioned before, we applied the classifier to all six preprocessed dataset options in the setting of 100 × 5-fold cross-validation SVM with linear kernel. The results of the analysis are presented in Table A1.1 in Additional file 1, while the ROC curves can be found in Figures A1.1–A1.6 in Additional file 1.

#### Decision tree

The classifier was applied to all datasets with 4 different depth limits: 2, 4, 8, and 16, with lower and upper limits being empirically determined to be too low and too high respectively for each dataset. The results of the analysis are presented in Table A2.1 in Additional file 2. Figures A2.1–A2.6 in Additional file 2 present ROC curves for each dataset and for each tree depth limit.

When using the decision tree classifier, the first preprocessing option (replacing time series with their last values) seems to be showing the best results (highest F1 score and overall accuracy). However, this preprocessing technique is the least valuable option for application in a real clinical setting, since the value sufficient for correct discrimination might be generated too late to be of any practical use. Depth limit of 4 usually exhibits the best performance with a single exception of the last preprocessing option.

### Influence of comorbidities

The final performance measurements are presented in Table 6. All the classifiers were applied to the dataset with ANN showing the best results.

**Table 6:** Performance evaluation with comorbidities

	Precision	Recall	F1 score	Accuracy	AUC
ANN	**0.8328**	0.7221	0.7734	0.8120	0.8922
SVM	0.7795	0.7864	0.7823	0.8054	0.8644
Decision tree	0.7982	**0.8152**	**0.8064**	**0.8261**	**0.8988**
Linear Regression	0.7934	0.8134	0.8031	0.8228	0.8926
Logistic Regression	0.7961	0.8115	0.8036	0.8238	0.8941

According to the table, ANN classifier produces the highest recall, F1 score, and accuracy of prediction, while SVM classifier shows the highest precision. Recall is a much more valuable measure for medical predictions, since in clinical settings it is much more important to detect as many patients having the condition as possible, rather than detecting some of them with higher precision. This means that ANN classifier is the most relevant for prediction of polyneuropathy.

## Discussion

We have made an exploratory study to research real-world evidences of the polyneuropathy development risks. Despite considering endpoint events on polyneuropathy we tried to make more general conclusion to understand what features are important for the prediction. For example, as revealed in the study blood sugar is one of the most important features. This real-world evidence can be used to set up a study for example on the influence of portable and wearable devices on the polyneuropathy risk management.

### Subclasses in diabetes population

As a result of the cluster analysis, several groups of patients were identified: younger men and women under 45 (clusters 4 and 3, the share of polyneuropathy is significantly lower), older men and women (clusters 1 and 2 are men, 0 are women, the share of polyneuropathy is significantly higher). The study of the peculiarities of clustering of demographic and laboratory indices under study revealed the following regularities:

1. Higher blood glucose (Glu_max) figures are associated with the development of polyneuropathy in older men.
2. The higher the age, the less the antiatherogenic potential decrease (HDLs decrease), and the higher the rate of renal excretion (creatinine increases). Both phenomena are associated with the development of polyneuropathy. The findings raise the question of rational hypolipidemic therapy, in particular, the safety endpoints of statins. Hematological changes associated with polyneuropathy do not depend on the patient’s gender. The share of lymphocytes increases with age, the share of neutrophils decreases (characteristics of the chronic inflammatory process), hematocrit increases and the concentration index, the mass content of hemoglobin, increases. The last two parameters can be associated with slowly growing water balance disorders, which is most often observed in patients receiving diuretics for a long time without sufficient control by the medical service. These abnormalities may be comorbidal to developing circulatory hypoxia.

### Correlation analysis and feature importance

The results of correlation analysis are of high clinical significance. Several models of pathophysiological reactions of the patients with diabetes mellitus were confirmed, in particular, the development of the inflammatory process based on the increase in the relative number of cells of the monocytic series, which reflects chronic inflammation.

Positive correlation of polyneuropathy presence with the patient’s age was also demonstrated. The mean platelet volume (MPV) has a positive correlation with glucoseuria and hyperglycemia. Patients with higher MPV values have larger and more active platelets, which may be important in predicting cardiovascular complications of diabetes mellitus [24]. Previously, researchers have provided evidence that a dependency between increased MPV and insufficient glycemic control [25], moreover, when observing patients receiving optimal treatment, evidence of a decrease in MPV following the normalization of glycaemia was demonstrated [26]. Thus, it can be concluded that there is a link between insufficient control of glycaemia and the development of progression of diabetes complications in the form of polyneuropathy. We have not found any serious evidence of this relationship in the literature. If we discuss the main features (indicators) affecting the development of polyneuropathy in diabetes, the decrease in the relative number of segmented neutrophils, which supports the postulate of the main contribution of monocyte cells in the process of inflammation development [26], as well as signs of unsatisfactory glycemic control, manifested in glucoseuria and hyperglycemia, become clinically important events.

### Sensitivity analysis

#### Artificial neural network classifier

The experiments have shown that the best performing dataset is the one where we represent time series as sets of their statistical characteristics. The result is very promising, despite this preprocessing technique not having any clear clinical interpretation behind it, because this option is the most easily expandable by including additional statistical characteristics of the time series (e.g. median or autocorrelation), and allows to utilize the information from the time series to the greatest extent, potentially including some temporal features which are not representable when using other preprocessing methods. Additionally, this option is easily optimizable since it has the largest number of features, some of which might appear to be redundant.

#### SVM classifier

The experiments showed that a linear kernel SVM classifier exhibits the highest predictive performance. As for the options of data preprocessing, replacing each time series with a set of respective statistical characteristics and with maximums (and minimum for haemoglobin) shows comparable results, and filling the missing data in from the medians shows the best results. High results from the last preprocessing option were expected given the known clinical interpretation of the values. Additional benefit of using this type of preprocessing is that it has lesser dimensionality, which reduces the computational cost of SVM application and therefore produces the result faster when employed on the same hardware.

#### Decision tree

**Fig. 4:**
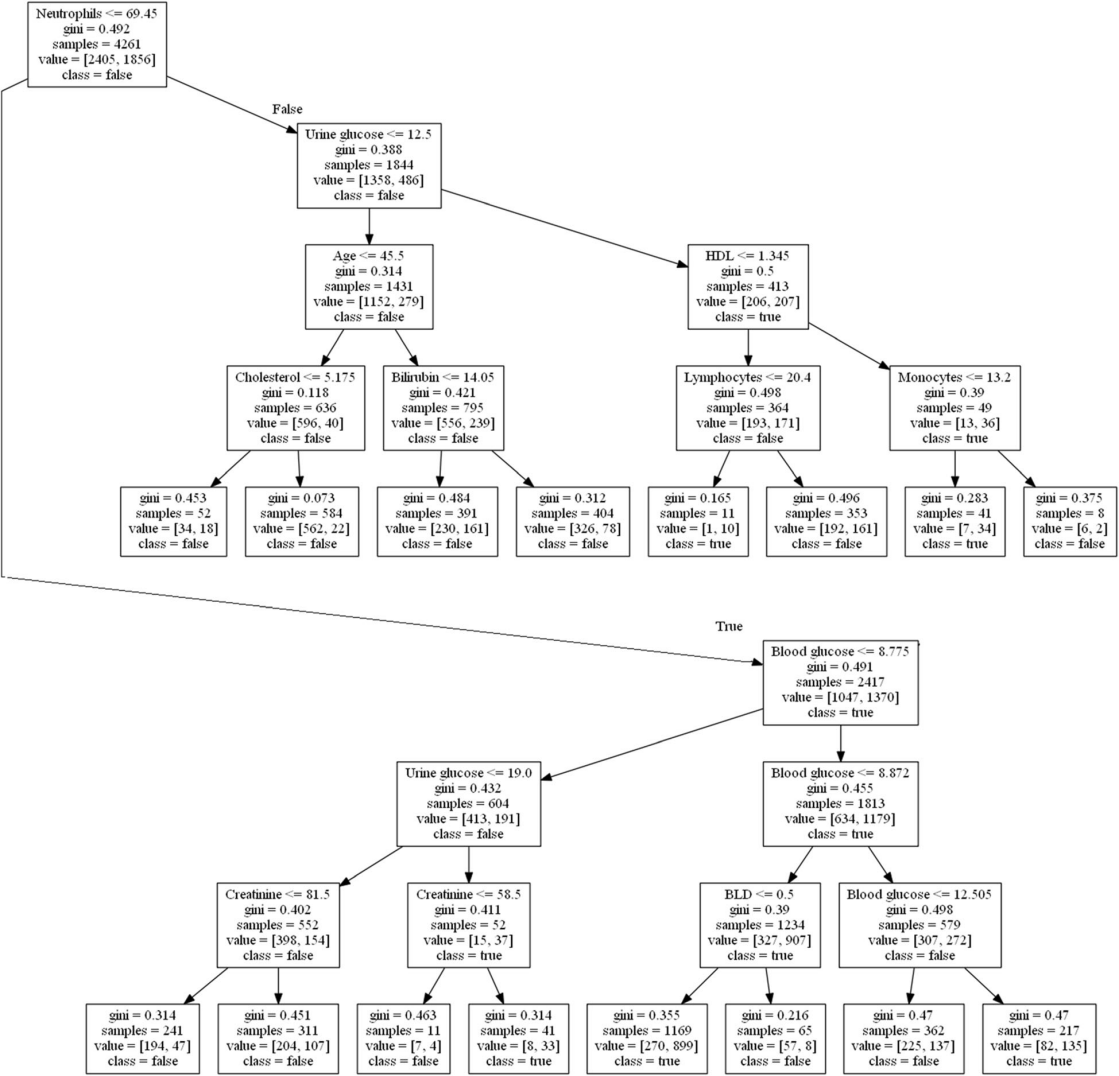
Decision tree for polyneuropathy prediction

The decision tree (see Fig. 4) shows the importance of the neutrophil level. For patients with high neutrophil levels, urine glucose levels and age are important. Patients with high levels of neutrophils and younger than 45 years with high cholesterol are at risk. The same patients older than 45 years with increased bilirubin also fall into the risk group. At normal neutrophil levels and normal blood and urine glucose levels, the risk factor is increased creatinine, which reflects kidney function. It is evident that various pathological mechanisms are leading to the emergence of polyneuropathy. The decision tree perfectly demonstrates these mechanisms: immunological processes, accompanied by the development of severe systemic inflammation and production of autoantibodies [27]. Therapeutic approaches can be based on these pathogenetic mechanisms.

As a result of the analysis of the “decision tree”, interesting results were obtained predicting the development of diabetic polyneuropathy in a patient with diabetes, based on routine laboratory methods and basic demographic data. From the point of view of this method of analysis, one of the key points is the relative number of neutrophils, and the development of polyneuropathy is associated with a decrease in the relative number of neutrophils, which is also confirmed by correlation analysis. Clinically, patients with diabetes mellitus have a systemic inflammatory process in the development of polyneuropathy, evidence of which was provided by sensitive specific markers (increased synthesis of proinflammatory cytokines, C-reactive protein, proliferation of macrophages).

The components of the common blood count test show a decrease in the relative number of neutrophils and an increase in the relative number of lymphocytes, and it is these parameters that are included in the decision tree for the detection of polyneuropathy in diabetes mellitus.

Other sensitive nodes of decision-making from the clinical point of view are the signs of uncompensated glucose metabolism (indicators: glucose in blood and urine) and the development of other complications associated with microangipathy (indicator: concentration of creatine in plasma, which is indirectly associated with developing nephropathy). Interestingly, the branch of the negative decision about the presence of polyneuropathy in patients with diabetes mellitus is based on the young age of the patient (under 45 years), low concentrations of total cholesterol and bilirubin, and the latter two indicators are associated with hypolipidemic therapy and administration of lipidic acid drugs by patients with a moderate hepatoprotective effect. All of this demonstrates that correct therapy of risk factors for the development and progression of concomitant lesions in diabetes can reduce the rate of development of diabetic polyneuropathy.

### Influence of comorbidities

The analysis shows that addition of the information about the diabetes comorbidities allows to further increase the model’s performance by 1–2% AUC, reaching 89.88% AUC for ANN classifier. Diabetic nephropathy and retinopathy appeared to be the best predictors across the board.

**Fig. 5:**
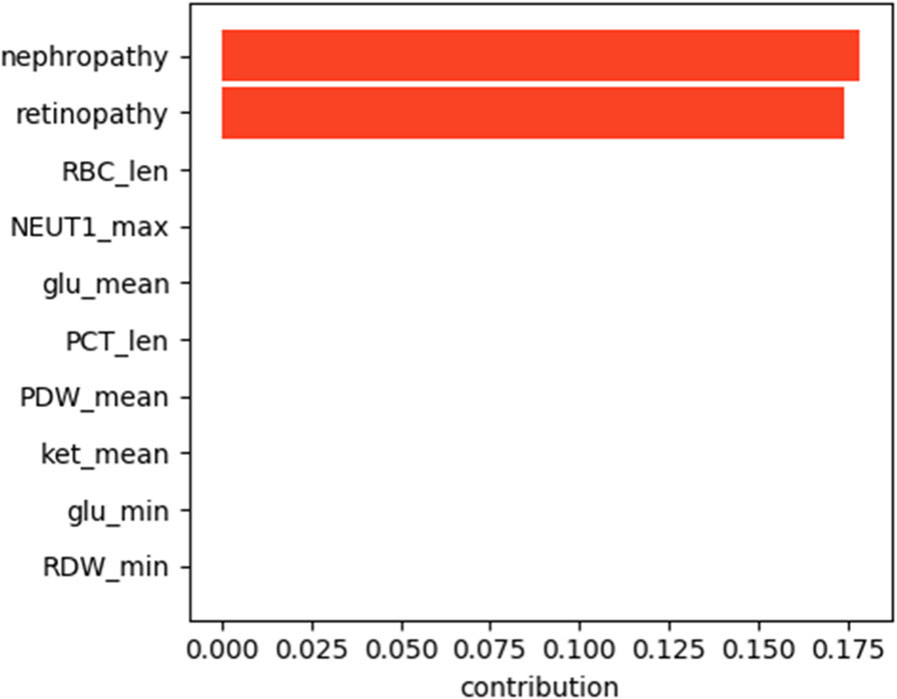
SVM interpretation

**Fig. 6:**
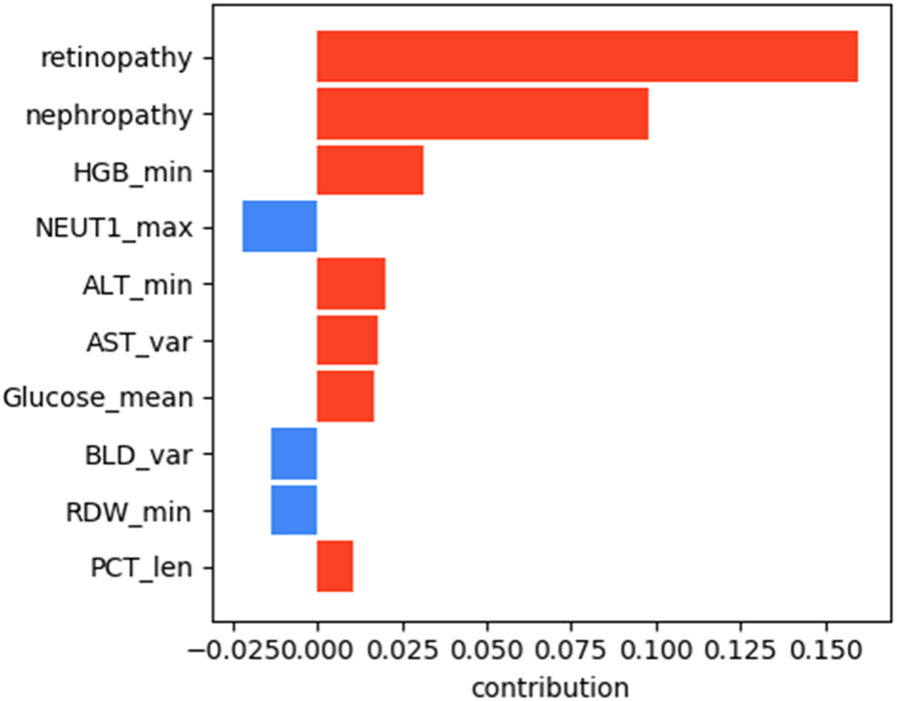
DT interpretation

**Fig. 7:**
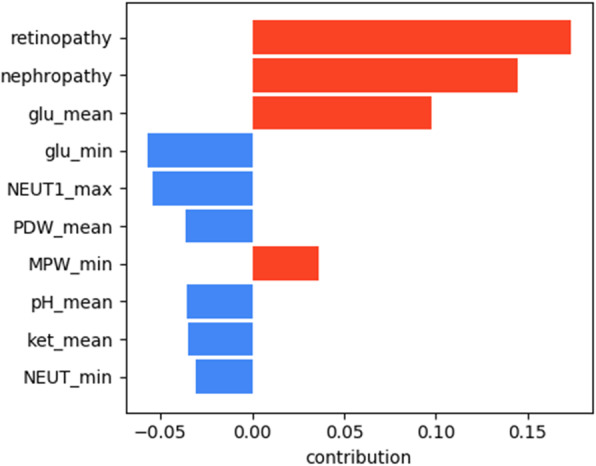
ANN interpretation

**Fig. 8:**
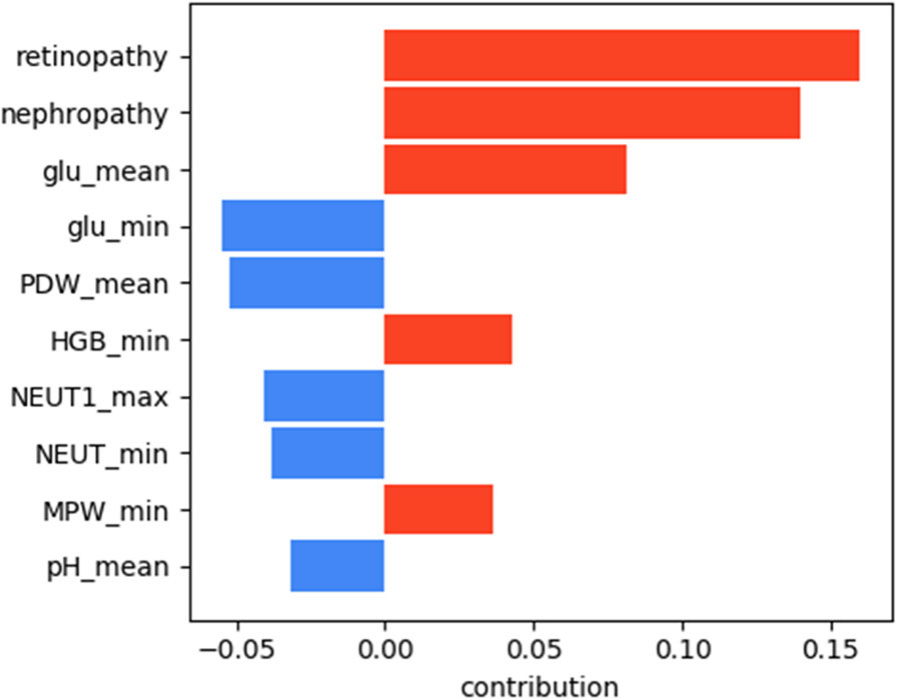
Logistic regression interpretation

The results of the interpretation obtained using LIME are presented in Figs. 5, 6, 7, 8 for all models except for linear regression.

**Fig. 9:**
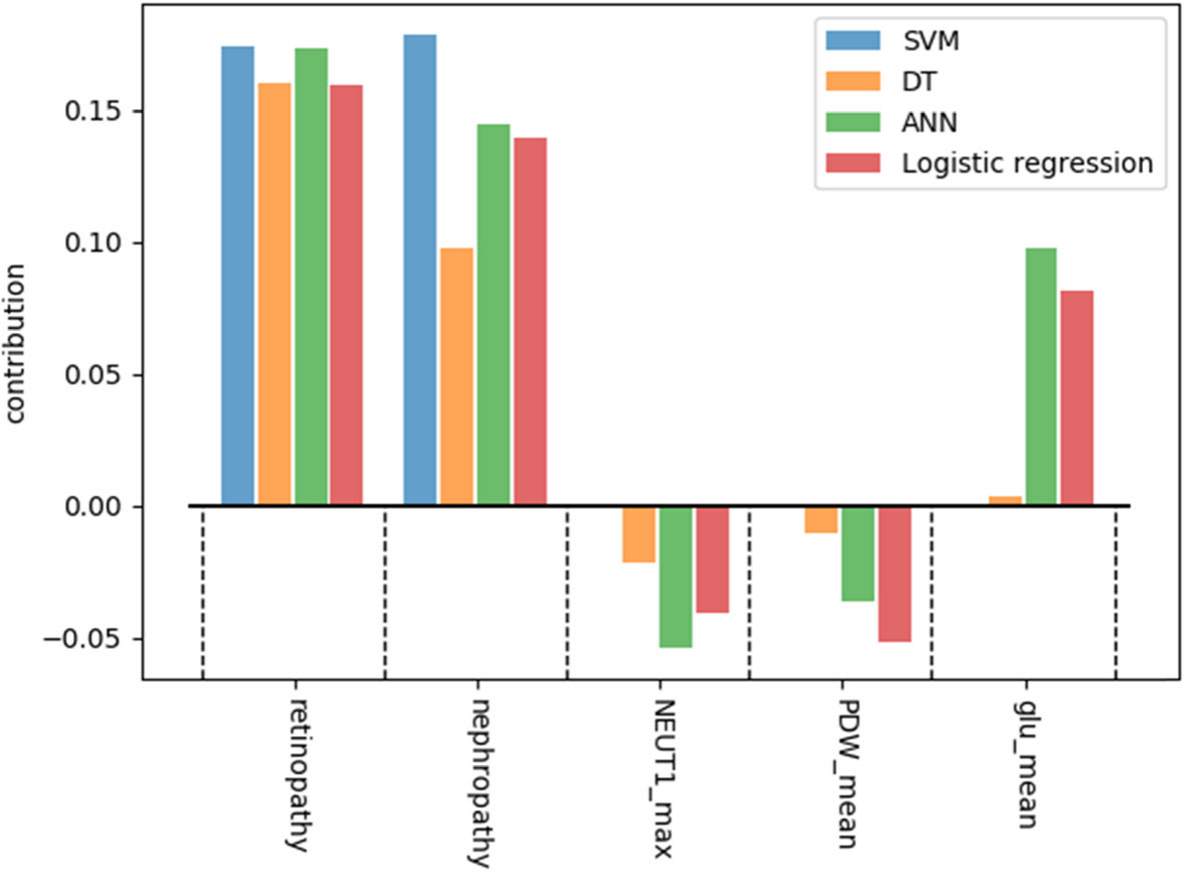
Interpretation summary

We can see that nephropathy and retinopathy have the highest contribution across all four classifiers, while other parameters vary. Figure 9 presents the summary of the analysis, displaying the most important features for every method.

The figure shows that high blood glucose levels also increase the chances of a patient having polyneuropathy, while high PDW (platelet distribution width) and NEUT1 (neutrophils concentration) tend to favour the opposite outcome. From the clinical point of view, the presence of retinopathy and nephropathy is certainly not the cause of polyneuropathy, they are all a consequence of metabolic disorders in diabetes mellitus or, more correctly, insulin resistance: one of the main elements of the development of diabetes mellitus. They should improve the quality of prognosis, because these pathological conditions usually develop faster than polyneuropathy and this coincides with the older age of patients. We can conclude that if a patient has polyneuropathy but no nephropathy or retinopathy, then diabetes becomes questionable as a cause of polyneuropathy.

### Study implications

Diabetic polyneuropathy leads to the development of refractory pain syndrome, which in some cases is poorly treated. The dissatisfaction with the available approaches to therapy is emphasized by a large number of recent studies aimed at finding new approaches. The commonly accepted marker for polyneuropathy is changes in corneal nerve structure from microscopy data [28]. Other methods of diagnostics are ultrasaund [29], Magnetic Resonance Imaging of nerves [30].

Meta-analysis of studies on the efficiency of different treatment methods compared to placebo has shown that many methods are no better than placebo [31]. There are no universally available and effective drugs to effectively control progression or prevent Diabetic Polyneuropathy [32].

Early diagnosis of diabetic polyneuropathy is difficult. General clinical methods allow to diagnose the disease already in its unfolded stage, which limits the possibility of early intervention.

Prevention is poorly developed, pathogenesis is complex, the role of individual factors is unclear (the degree of compensation for diabetes, microangiopathy, systemic inflammation, kidney dysfunction, macroangiopathy, possible others).

The use of modern methods of analysis of large amounts of data accumulated in real practice will allow to better study the factors associated with the development of diabetic polyneuropathy, and in different groups of patients will allow to predict the probability of this complication, to formulate hypotheses of studies on preventive measures and make a step towards significant improvement of control over the development of this complication.

We can make the following key conclusion from our study. Clinical data collected in the framework of routine examinations of diabetes patients and accumulated in medical information systems can be used to identify (by means of machine learning methods) patients with high risk of diabetic polyneuropathy development.

The analysis of factors associated with the development of diabetic polyneuropathy allows to assume the complex character of the given complication, the genesis of which is contributed by unsatisfactory control of glycemia, systemic inflammation, renal dyslipidemia, dyslipidemia, macroangiopathy, and different factors have different significance for different subgroups of diabetes patients (depending on sex, age, specific course of the disease, existing complications). Development and clinical adaptation of prognostic models will allow to improve detection of patients with high risk of diabetic polyneuropathy and to implement the preventive approach and early interventions, however, it requires prospective clinical studies.

The main problem that this study may solve is the delayed diagnosis of polyneuropathy. Doctors at all levels of medical care (polyclinics and hospitals) need additional tools to help diagnose diabetic polyneuropathy, distinguish this variant of polyneuropathy from others, prescribe treatment in time, and control the process of treatment. In fact, there are several stages in the development of polyneuropathy. At the first stage, there is a functional readjustment of the peripheral nervous system: early changes in sensitivity (more often hyperesthesia, increased sensitivity distally: hands and feet, as a symptom of irritation, a replacement of loss of balance of function), but in this case, the treatment (pathogenetic, aimed at the mechanisms of pathological processes) can be successful and establish a close to physiological balance of sensitivity. Care at this stage is already less effective, and there is an increased need for symptomatic therapy (i. e., only symptomatic treatment) for pain management. In the last stage of the process, one can observe the development of significant disturbances of homeostasis of skin structures, subcutaneous tissue, immune system, and blood supply system. Even insignificant effects (abrasions, grown nail) can lead to serious consequences. The developed classification algorithm, which can be transformed into a scale of risk assessment of polyneuropathy development, its progression, and outcomes (ideally) can affect the rate of development of polyneuropathy. Such a tool will reduce the consumption of drugs for the treatment of pain syndrome, reduce the likelihood of nontraumatic amputation due to diabetic polyneuropathy, and improve the quality of life of a patient with diabetic polyneuropathy.

In such complicated situation, an important methodological issue arises in selection of proper predictive models. The applicability of a model may be considered from two key points of view. First, basic performance measures enable the selection of a model. Our study shows that in various conditions different models have different performance with different measures. Moreover, clinical context brings additional requirements to the priority of various quality measures. E. g., in many medical cases, false negative prediction is a crucial problem while false positive prediction is considered a minor issue. Considering these aspects and having multiple predictive models available in modern libraries, an idea of automatic selection of the best model seems to be promising. Such solutions could bring intelligent management and a next level of automation into the systems based on predictive modelling. Second, an important aspect of predictive models is interpretability. The level of interpretability is related to the level of trust, proof, interactivity, and automation in predictive modelling. Within the considered model, DT and linear regression could be treated as more interpretable, while ANN is obviously a less interpretable one. Still, currently, interpretable machine learning [33] has become an important direction in machine learning. That enables application of modern tools like LIME, SHAP, etc. in practical solutions. Moreover, proper interpretation may resolve the problem of automation as it provides explanation of both model structure and modelling result.

In comparison to the previous studies [11, 12, 18, 19] our research makes a comprehensive comparison of different machine learning methods using a well-structured, large enough and balanced dataset applied to various decision support tasks in the field of diabetes complications. We did not focus on one machine leaning method, but searched for the most efficient method for each of the study tasks. Our study has achieved a better accuracy than more traditional research on risk factors for diabetic polyneuropathy based on logistic regression. For example [34] achieved 82.8% of sensitivity and specificity of 61.9%. The study [35] has reported 76% accuracy. The higher performance of our models in comparison to the state of the art can be explained by a larger number of predictors and inclusion of comorbidities. In comparison to the models in [34, 35] we did not use diabetes specific predictors. Instead we included common laboratory test results, so our models can be applied also to the patients not yet diagnosed with diabetes.

So, the results of the work provide an overview of the performance of different models that can be useful for developers of clinical decision support systems.

## Conclusion

Clustering shows differences within patients with diabetes mellitus. Decision trees demonstrate pathophysiological mechanisms. It is possible to identify under what conditions the current risks of polyneuropathy play the most significant role. Based on the risk models, it is possible to plan a personal therapy appointment to prevent the development of complications. The obtained results can be used as predictive components for clinical decision support systems. Early calculation of patient risks will allow prescribing neuroprotective therapy for high-risk patients. This research is in progress. A similar method of extracting knowledge is possible for other diseases.

It was discovered that inclusion of two expressions, namely “nephropathy” and “retinopathy”, allows to increase the performance, achieving up to 79.82% precision, 81.52% recall, 80.64% F1 score, 82.61% accuracy, and 89.88% AUC using the neural network classifier.

Additionally, different models showed different results in terms of interpretation significance. Random forest confirmed that the most important risk factors for polyneuropathy are increased neutrophil level, meaning the presence of inflammation in the body. Linear models show linear dependencies of the presence of polyneuropathy on blood glucose levels, which is confirmed by the clinical interpretation of the importance of blood glucose control. Neural networks demonstrate the contribution of comorbidities (nephropathy and retinopathy) to the development of polyneuropathy.

The general conclusion from this study supports the hypothesis that different models need to be applied for different interpretation purposes. Depending on whether one needs to identify pathophysiological mechanisms for a prospective study or identify early or late predictors, the choice of model will vary.

Our results indicate the feasibility of creating a statistical model for predicting the development of diabetic polyneuropathy in patients with diabetes mellitus for prescribing or adjusting therapeutic strategies to reduce the risk of peripheral nerve damage, which should improve the quality of life of patients with diabetes.

## Study limitation

The study has the following limitation:

It was possible that our patients were not representative of all patients with diabetes, since this study was performed in one center. The patients included in the study are originated from a North-West region of Russia, mainly Saint-Petersburg and the Leningrad region. A multicenteric study could potentially result in better performing models. However, the number of cases and the balanced data set allows us to presume that the developed models can be used for a wide range of cases. We would recommend that the future studies include different population in terms of geography and race.

The models were implemented considering among other parameters only comorbidities, namely nephropathy and retinopathy as the available data set did not contain information on other comorbidities. Although these two are the most common ones, considering other comorbidities can potentially improve the models’ performance.

## Supplementary information

**Additional file 1.** SVM classifier results.

**Additional file 2.** DT classifier results.

**Additional file 3.** ANN classifier results.

**Additional file 4.** LR-based classifier results.

**Additional file 5.** Logistic regression classifier results.

## Data Availability

The datasets used and/or analysed during the current study are available from the corresponding author on reasonable request.

## Abbreviations

ANN: Artificial neuron network
DT: Decisions tree
AUC: Area under the Curve
ROC: Receiver operating characteristic curve
PDW: Platelet distribution width)
SVM: Support vector machine
DPN: Diabetic polyneuropathy
LR: Linear regression
HGB: Hemoglobin
LEU: Leukocytes
PLT: Platelets
MPW: Mean platelet volume
MCH: Mean cell hemoglobin
NEUT: Neutrophils
MCV: Mean corpuscular volume
PCT: Procalcitonin
RDW: Red blood cell distribution width
ALT: Alanine transaminase
PDW: Platelet distribution width
HDL: High-density lipoprotein
AST: Aspartate aminotransferase
WBC: White blood count
RBC: Red blood cell count
HCT: Hematocrit
LDL: Low-density lipoproteins
BLD: Blood in urine
