# Supplementary material for: Identification of risk factors for patients with diabetes: diabetic polyneuropathy case study
Source: BMC Med Inform Decis Mak. 2020 Aug 24;20:201. doi: 10.1186/s12911-020-01215-w (PMC7444272; doi:10.1186/s12911-020-01215-w)
Supplement: Supplementary file 1 — Additional file 1. SVM classifier results. [file 12911_2020_1215_MOESM1_ESM.docx]

# APPENDIX 1. SVM classifier results

Table A1.1 – SVM performance results

| Kernel | Linear | Polynomial | Sigmoid | Rbf |
| --- | --- | --- | --- | --- |
| Series replaced with the last values; missing values filtered out | | | | |
| Precision | 0.6689 | 0.0004 | 0.6796 | 0.6749 |
| Recall | 0.6477 | 0.0000 | 0.5941 | 0.6280 |
| F1-score | 0.6579 | 0.0000 | 0.6338 | 0.6504 |
| Accuracy | 0.7063 | 0.5635 | 0.7006 | 0.7056 |
| Series replaced with the last values; missing values filled in from medians | | | | |
| Precision | 0.6908 | 0.0000 | 0.7241 | 0.7006 |
| Recall | 0.6481 | 0.0000 | 0.4584 | 0.5234 |
| F1-score | 0.6686 | 0.0000 | 0.5607 | 0.5920 |
| Accuracy | 0.7227 | 0.5682 | 0.6904 | 0.6977 |
| Series replaced with the set of statistical characteristics; missing values filtered out | | | | |
| Precision | 0.7228 | 0.0130 | 0.7372 | 0.7265 |
| Recall | 0.6523 | 0.0000 | 0.5878 | 0.6246 |
| F1-score | 0.6856 | 0.0001 | 0.6538 | 0.6715 |
| Accuracy | 0.7391 | 0.5637 | 0.7286 | 0.7335 |
| Series replaced with the set of statistical characteristics; missing values filled in from medians | | | | |
| Precision | 0.7366 | 0.0910 | **0.8036** | 0.7676 |
| Recall | **0.6702** | 0.0002 | 0.2847 | 0.5235 |
| F1-score | **0.7017** | 0.0004 | 0.4186 | 0.6179 |
| Accuracy | **0.7541** | 0.5682 | 0.6609 | 0.7254 |
| Series replaced with maximums; missing values filtered out | | | | |
| Precision | 0.7192 | 0.0000 | 0.7245 | 0.7260 |
| Recall | 0.6417 | 0.0000 | 0.6128 | 0.6271 |
| F1-score | 0.6781 | 0.0000 | 0.6638 | 0.6728 |
| Accuracy | 0.7343 | 0.5636 | 0.7293 | 0.7340 |
| Series replaced with maximums; missing values filled in from medians | | | | |
| Precision | 0.7254 | 0.0000 | 0.7510 | 0.7130 |
| Recall | 0.6691 | 0.0000 | 0.5904 | 0.6419 |
| F1-score | 0.6960 | 0.0000 | 0.6609 | 0.6671 |
| Accuracy | 0.7477 | 0.5681 | 0.7385 | 0.7237 |

| 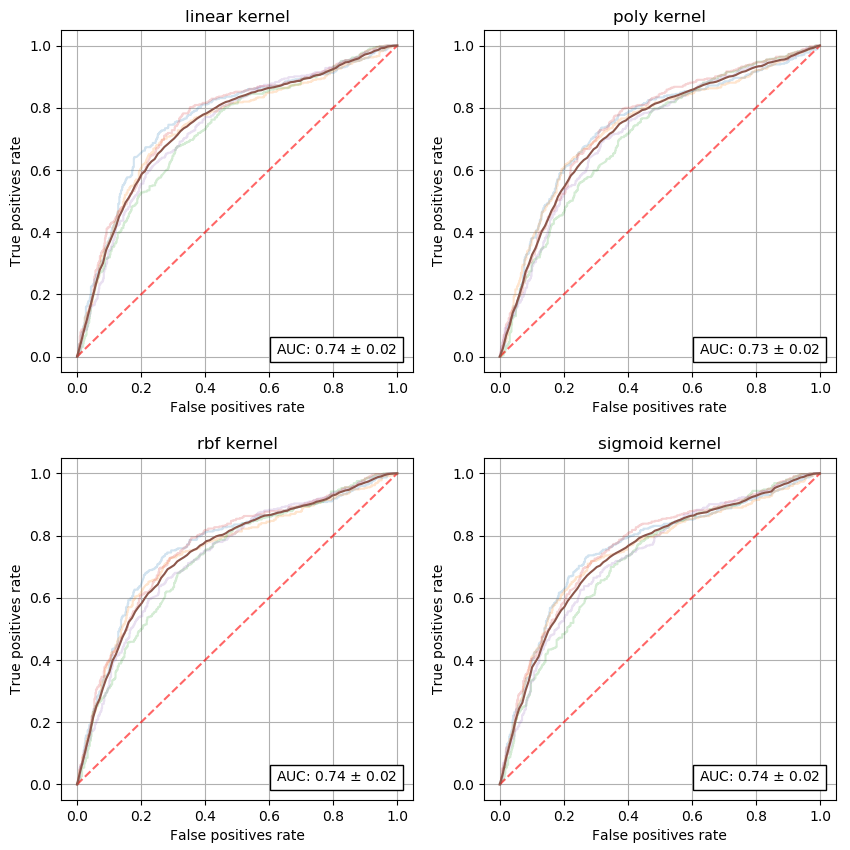 | 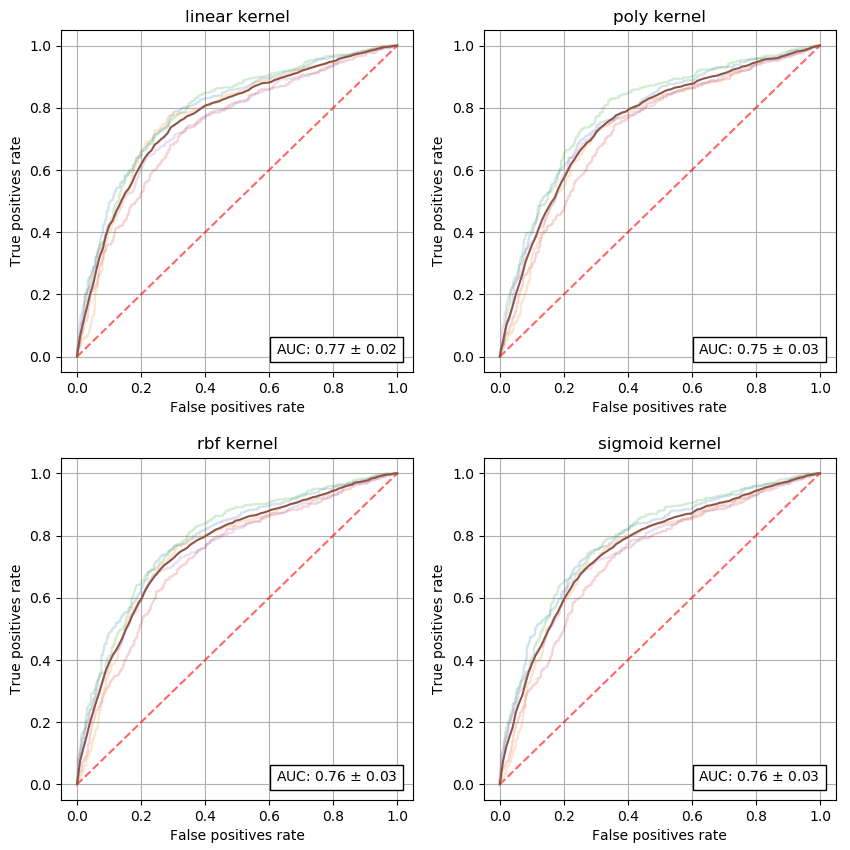 |
| --- | --- |
| Figure A1.1 – SVM ROC for file with series replaced with last values, missing data filtered out | Figure A1.2 – SVM ROC for file with series replaced with last values, missing data filled in |
| 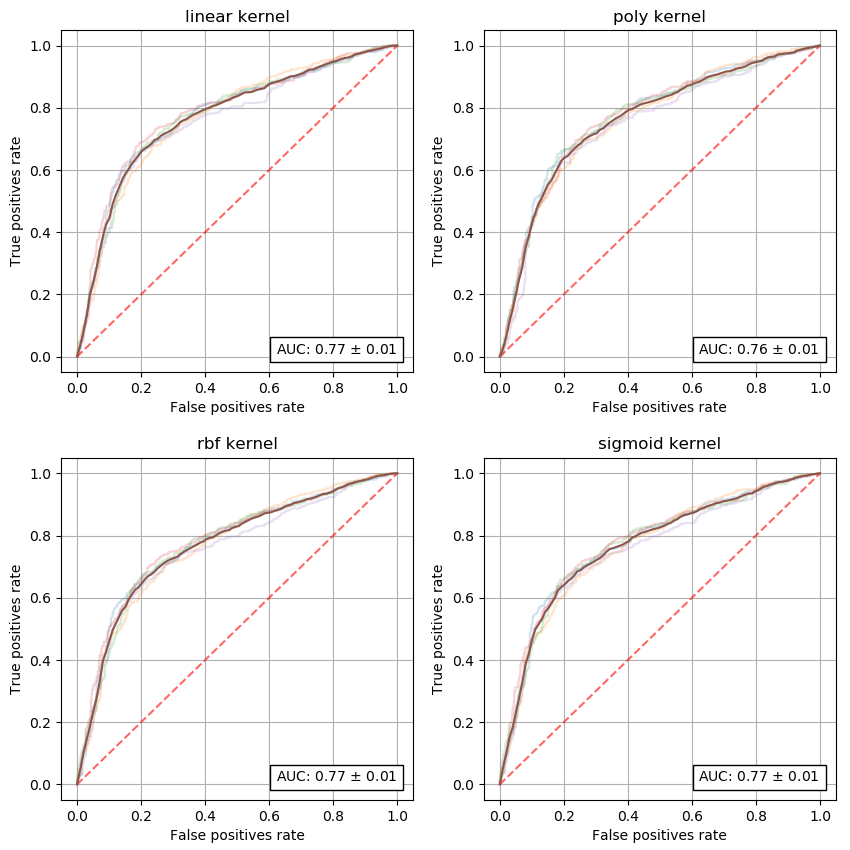 | 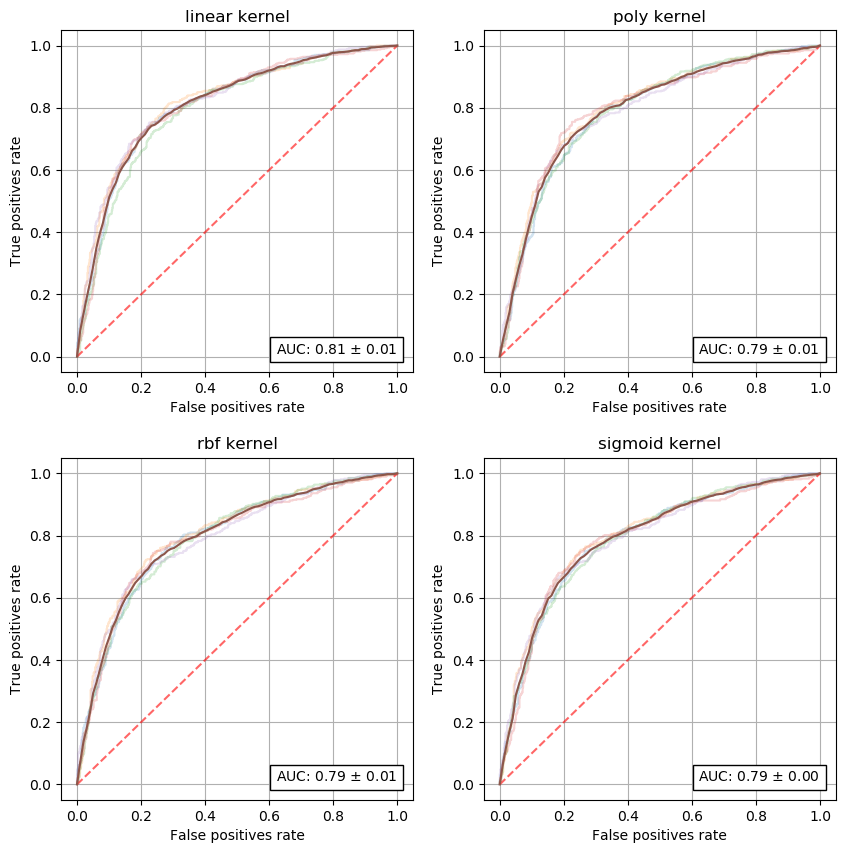 |
| Figure A1.3 – SVM ROC for file with series replaced with stats, missing data filtered out | Figure A1.4 – SVM ROC for file with series replaced with stats, missing data filled in |

| 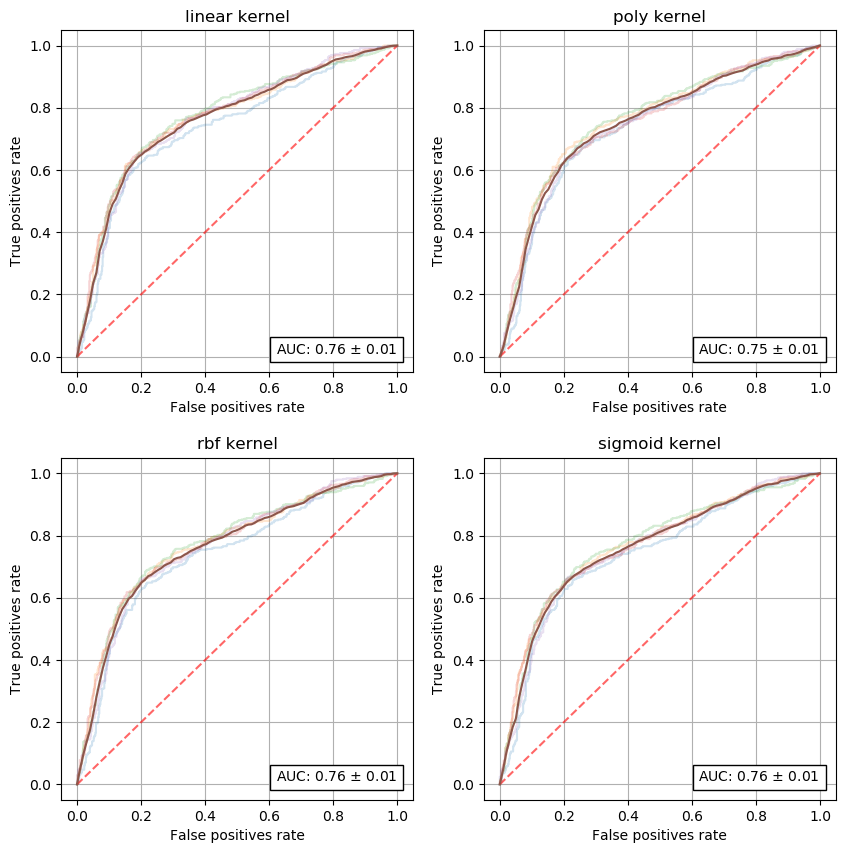 | 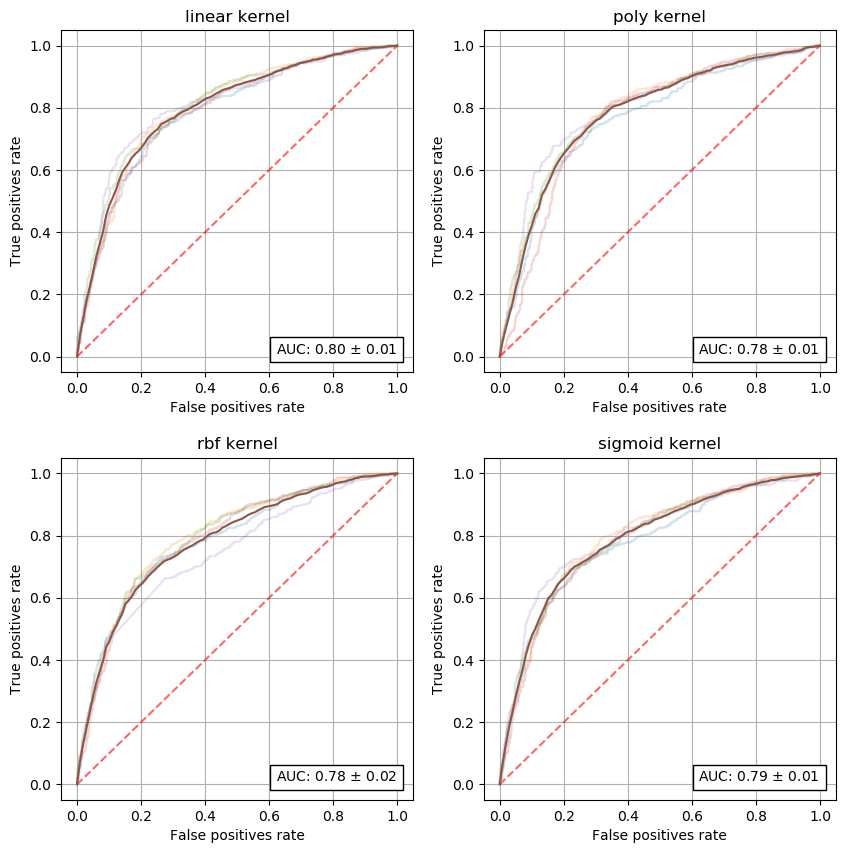 |
| --- | --- |
| Figure A1.5 – SVM ROC for file with series replaced with maximums, missing data filtered out | Figure A1.6 – SVM ROC for file with series replaced with maximums, missing data filled in |
