# Supplementary material for: Identification of risk factors for patients with diabetes: diabetic polyneuropathy case study
Source: BMC Med Inform Decis Mak. 2020 Aug 24;20:201. doi: 10.1186/s12911-020-01215-w (PMC7444272; doi:10.1186/s12911-020-01215-w)
Supplement: Supplementary file 2 — Additional file 2. DT classifier results. [file 12911_2020_1215_MOESM2_ESM.docx]

# APPENDIX 2. DT classifier results

Table A2.1 – DT performance results

| maximal depth | 2 | 4 | 8 | 16 |
| --- | --- | --- | --- | --- |
| Series replaced with the last values; missing values filtered out | | | | |
| Precision | 0.6139 | 0.6276 | 0.6264 | 0.5803 |
| Recall | 0.5929 | 0.6312 | 0.6265 | 0.5917 |
| F1-score | 0.6018 | 0.6280 | 0.6258 | 0.5857 |
| Accuracy | 0.6589 | 0.6750 | 0.6737 | 0.6351 |
| Series replaced with the last values; missing values filled in from medians | | | | |
| Precision | 0.7236 | 0.7244 | 0.6967 | 0.6397 |
| Recall | 0.6319 | 0.6407 | 0.6575 | 0.6566 |
| F1-score | 0.6745 | 0.6794 | 0.6761 | 0.6478 |
| Accuracy | 0.7368 | **0.7391** | 0.7282 | 0.6920 |
| Series replaced with the set of statistical characteristics; missing values filtered out | | | | |
| Precision | 0.6720 | 0.6977 | 0.6537 | 0.6016 |
| Recall | 0.5232 | 0.6036 | 0.6307 | 0.6238 |
| F1-score | 0.5867 | 0.6465 | 0.6414 | 0.6122 |
| Accuracy | 0.6798 | 0.7125 | 0.6927 | 0.6556 |
| Series replaced with the set of statistical characteristics; missing values filled in from medians | | | | |
| Precision | 0.6431 | 0.7203 | 0.6923 | 0.6452 |
| Recall | 0.5697 | 0.6142 | **0.6693** | 0.6623 |
| F1-score | 0.5986 | 0.6618 | **0.6801** | 0.6534 |
| Accuracy | 0.6747 | 0.7296 | 0.7285 | 0.6969 |
| Series replaced with maximums; missing values filtered out | | | | |
| Precision | **0.7356** | 0.7016 | 0.6641 | 0.5949 |
| Recall | 0.4643 | 0.6070 | 0.6230 | 0.6122 |
| F1-score | 0.5688 | 0.6500 | 0.6423 | 0.6031 |
| Accuracy | 0.6933 | 0.7153 | 0.6976 | 0.6489 |
| Series replaced with maximums; missing values filled in from medians | | | | |
| Precision | 0.6989 | 0.6997 | 0.6850 | 0.6358 |
| Recall | 0.5106 | 0.6144 | 0.6610 | 0.6458 |
| F1-score | 0.5844 | 0.6528 | 0.6722 | 0.6417 |
| Accuracy | 0.6905 | 0.7187 | 0.7220 | 0.6877 |

| 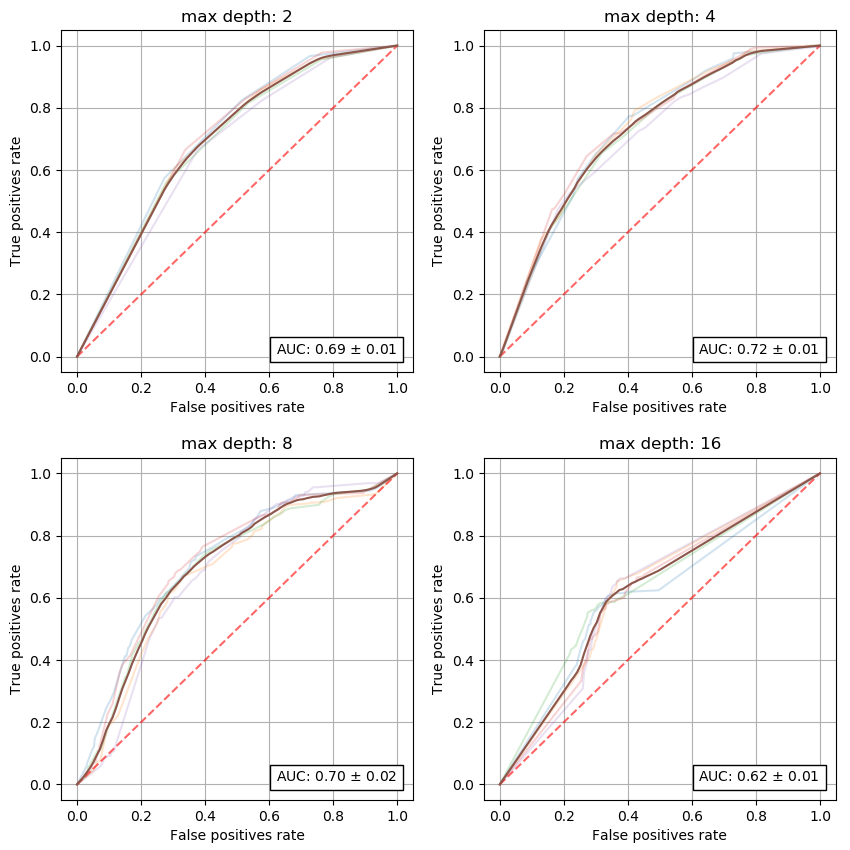 | 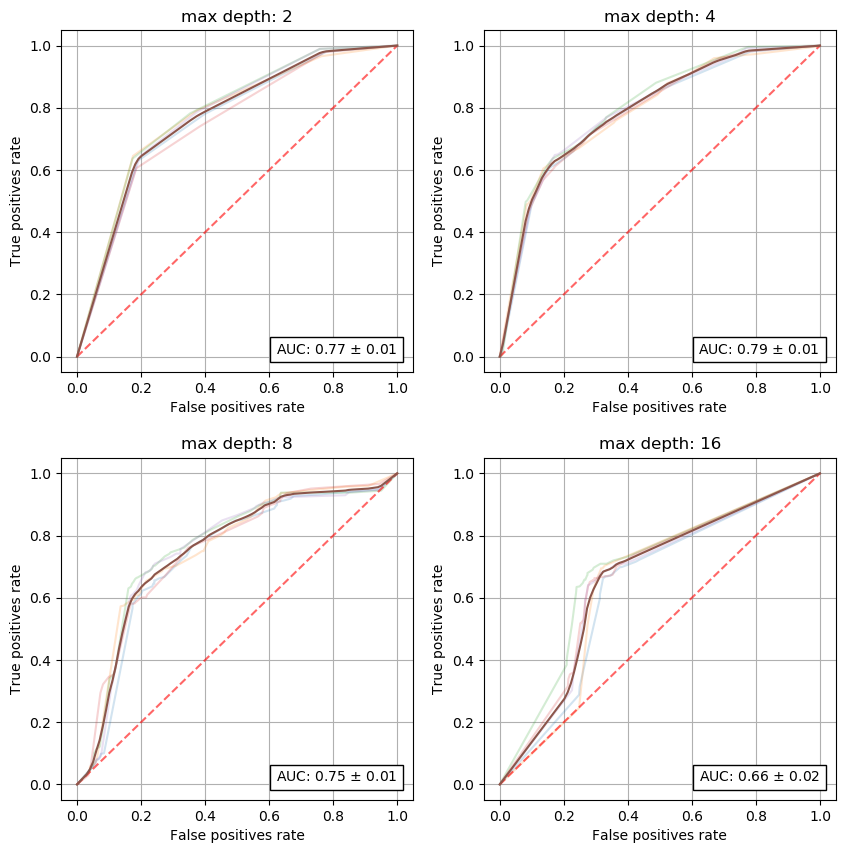 |
| --- | --- |
| Figure A2.1 – DT ROC for file with series replaced with last values, missing data filtered out | Figure A2.2 – DT ROC for file with series replaced with last values, missing data filled in |
| 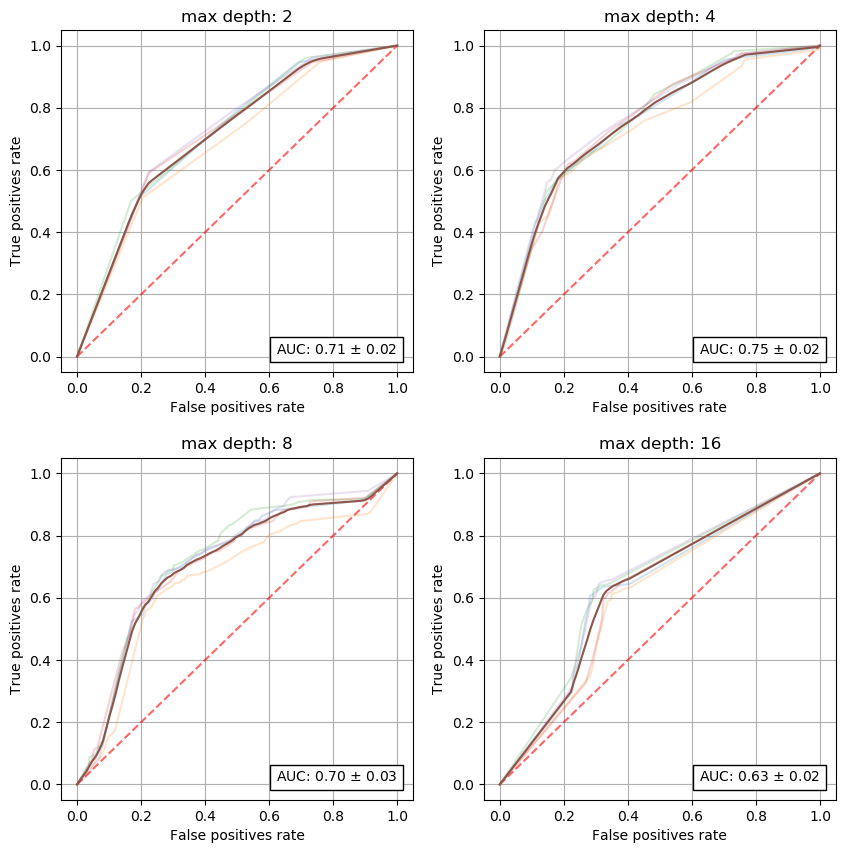 | 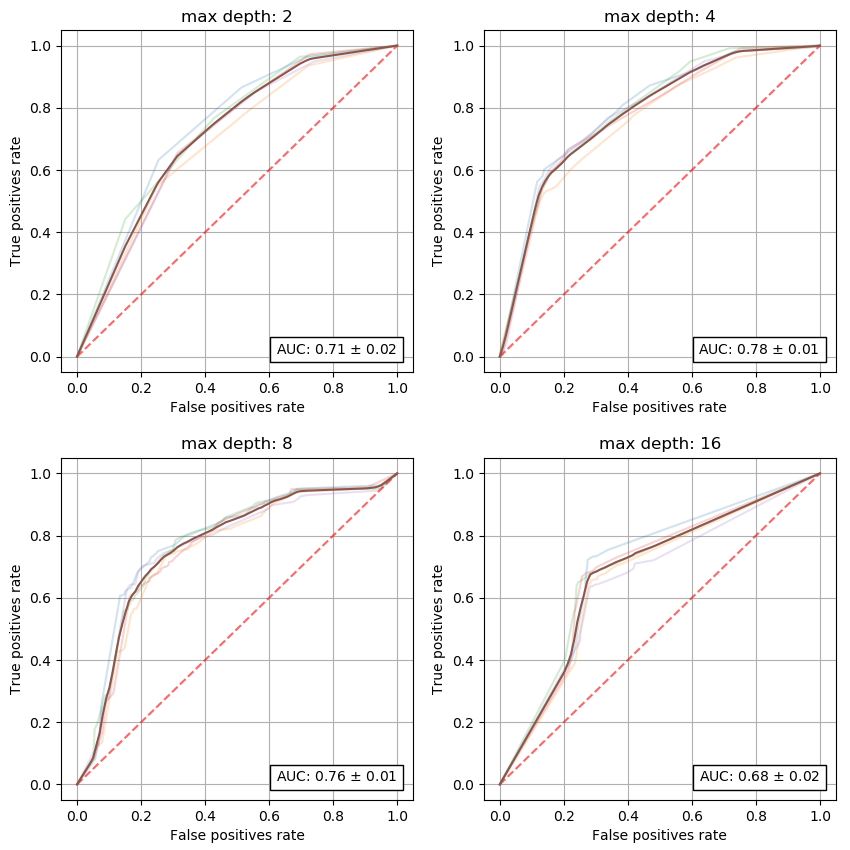 |
| Figure A2.3 – DT ROC for file with series replaced with stats, missing data filtered out | Figure A2.4 – DT ROC for file with series replaced with stats, missing data filled in |
| 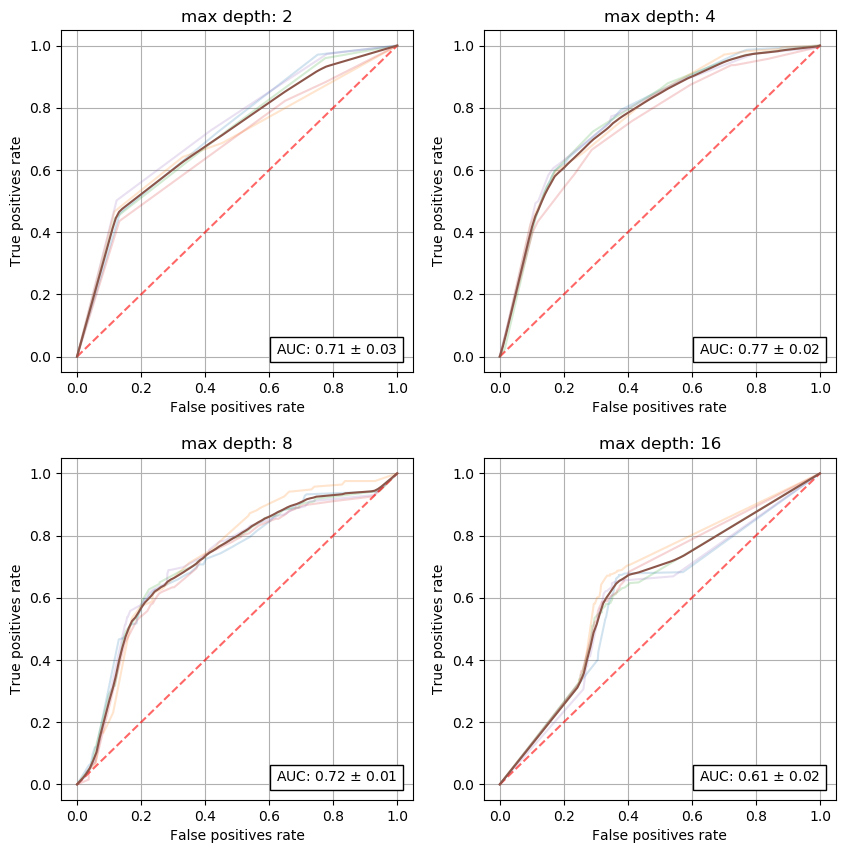 | 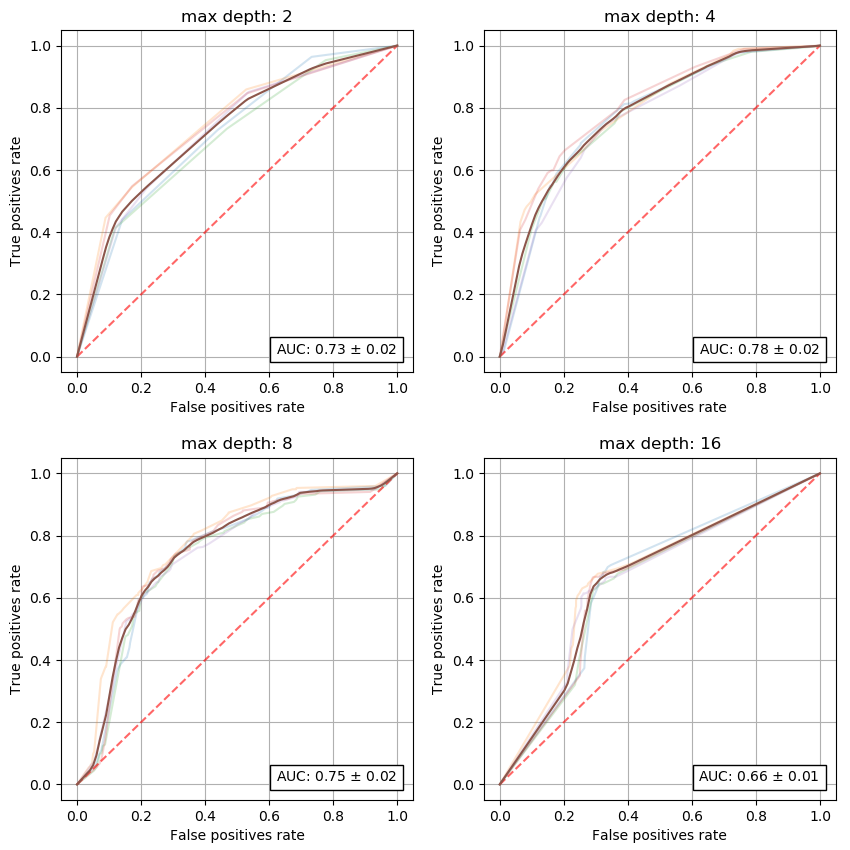 |
| Figure A2.5 – DT ROC for file with series replaced with maximums, missing data filtered out | Figure A2.6 – DT ROC for file with series replaced with maximums, missing data filled in |
