# Supplementary material for: Identification of risk factors for patients with diabetes: diabetic polyneuropathy case study
Source: BMC Med Inform Decis Mak. 2020 Aug 24;20:201. doi: 10.1186/s12911-020-01215-w (PMC7444272; doi:10.1186/s12911-020-01215-w)
Supplement: Supplementary file 3 — Additional file 3. ANN classifier results. [file 12911_2020_1215_MOESM3_ESM.docx]

# APPENDIX 3. ANN classifier results

| 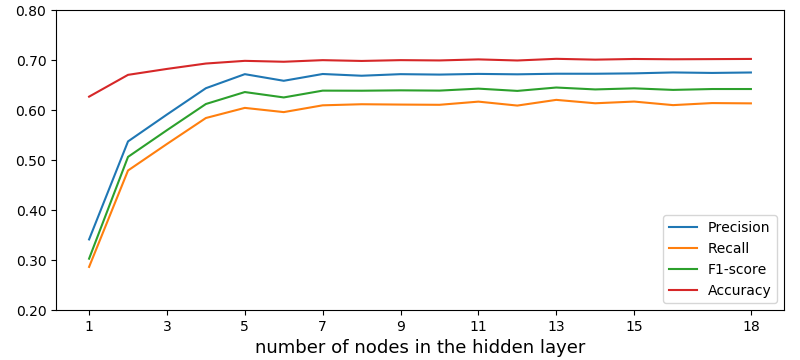 |
| --- |
| Figure A3.1 – ANN sensitivity analysis. Series replaced with the last values; missing values filtered out  Max F1 score of 0.6450 is achieved at 13 nodes in the hidden layer |
| 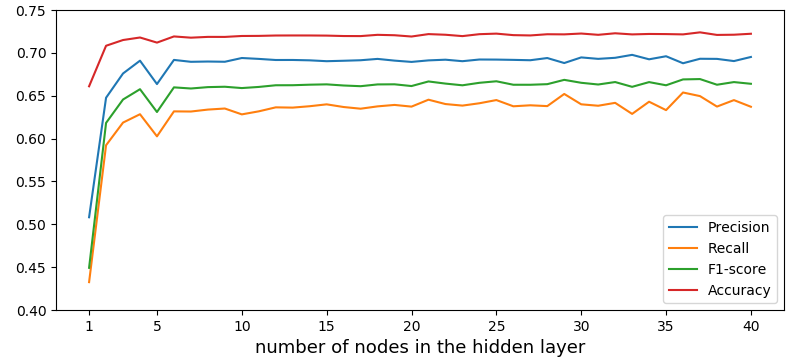 |
| Figure A3.2 – ANN sensitivity analysis. Series replaced with the last values; missing values filled in  Max F1 score of 0.6694 is achieved at 37 nodes in the hidden layer |
| 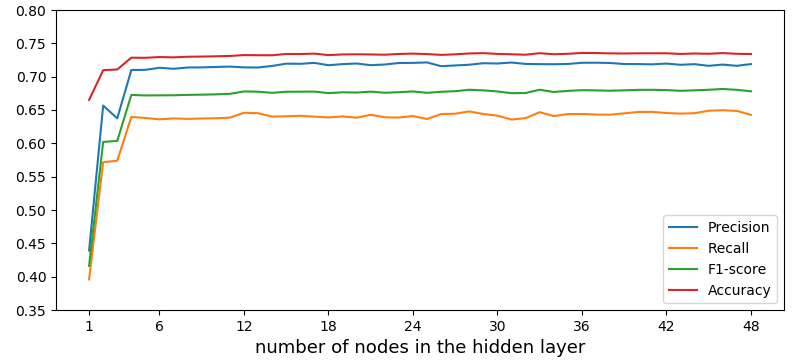 |
| Figure A3.3 – ANN sensitivity analysis. Series replaced with the stats; missing values filtered out  Max F1 score of 0.6815 is achieved at 46 nodes in the hidden layer |
| 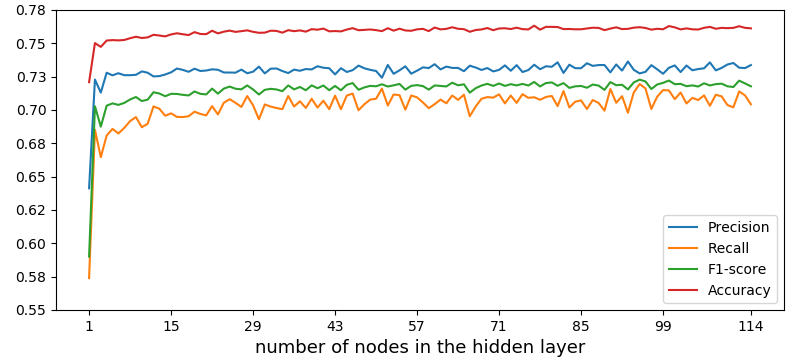 |
| Figure A3.4 – ANN sensitivity analysis. Series replaced with the stats; missing values filled in  Max F1 score of 0.7228 is achieved at 95 nodes in the hidden layer |
| 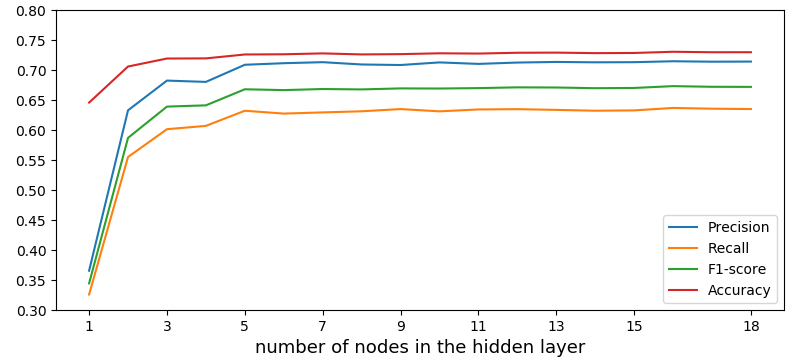 |
| Figure A3.5 – ANN sensitivity analysis. Series replaced with maximums; missing values filtered out  Max F1 score of 0.6731 is achieved at 16 nodes in the hidden layer |
| 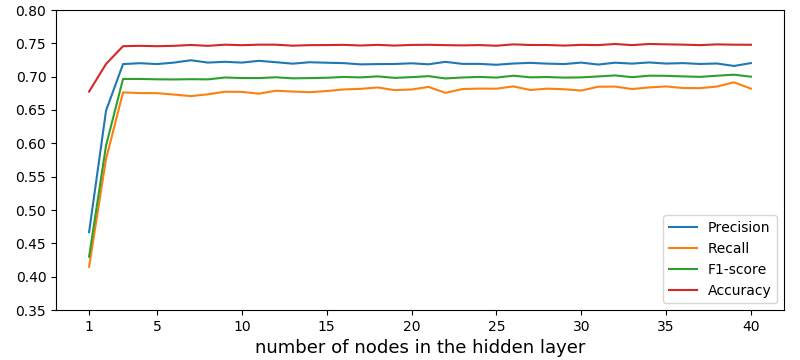 |
| Figure A3.6 – ANN sensitivity analysis. Series replaced with maximums; missing values filled in  Max F1 score of 0.7030 is achieved at 39 nodes in the hidden layer |

| 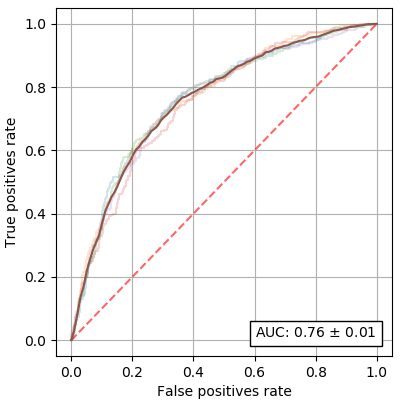 | | | | 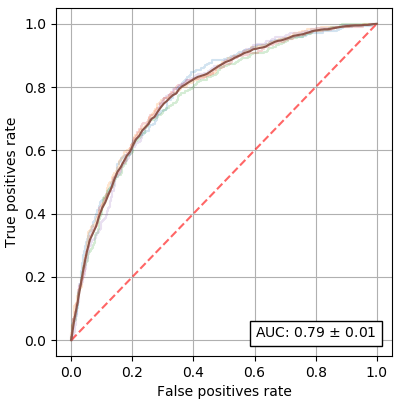 | | |
| --- | --- | --- | --- | --- | --- | --- |
|  | Figure A3.7 – ANN ROC for file with series replaced with last values, missing data filtered out | |  |  | Figure A3.8 – ANN ROC for file with series replaced with last values, missing data filled in |  |
| 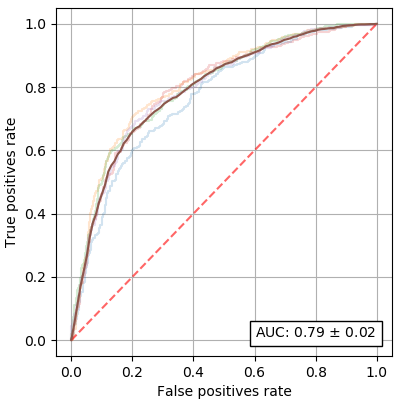 | | | | 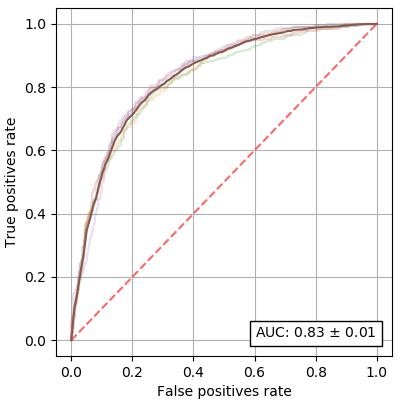 | | |
|  | Figure A3.9 – ANN ROC for file with series replaced with the stats, missing data filtered out | |  |  | Figure A3.10 – ANN ROC for file with series replaced with the stats, missing data filled in |  |
| 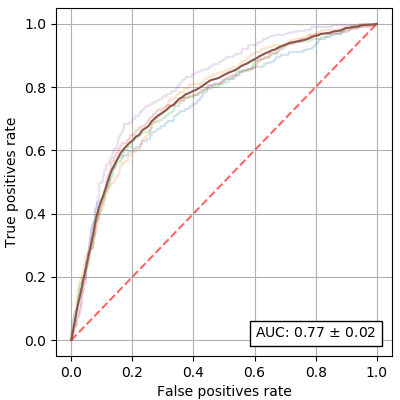 | | | | 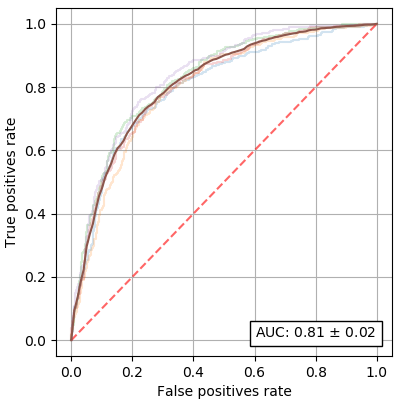 | | |
|  | Figure A3.11 – ANN ROC for file with series replaced with maximums, missing data filtered out |  | |  | Figure A3.12 – ANN ROC for file with series replaced with maximums, missing data filled in |  |
