# Supplementary material for: Identification of risk factors for patients with diabetes: diabetic polyneuropathy case study
Source: BMC Med Inform Decis Mak. 2020 Aug 24;20:201. doi: 10.1186/s12911-020-01215-w (PMC7444272; doi:10.1186/s12911-020-01215-w)
Supplement: Supplementary file 5 — Additional file 5. Logistic regression classifier results. [file 12911_2020_1215_MOESM5_ESM.docx]

# APPENDIX 5. Logistic regression classifier results

| 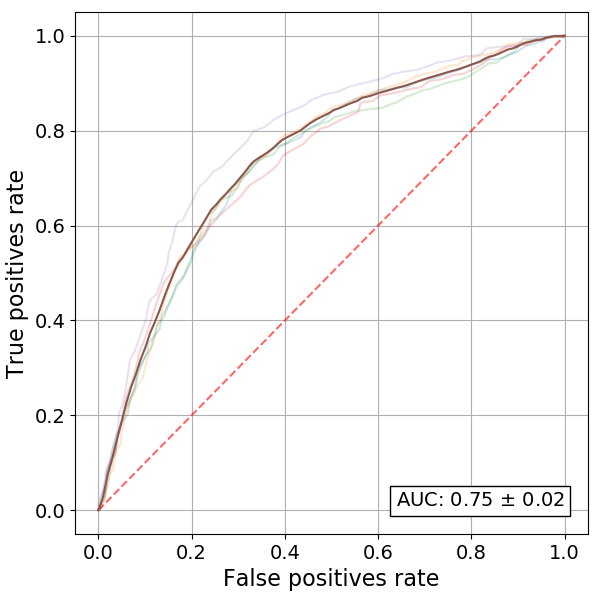 | | | 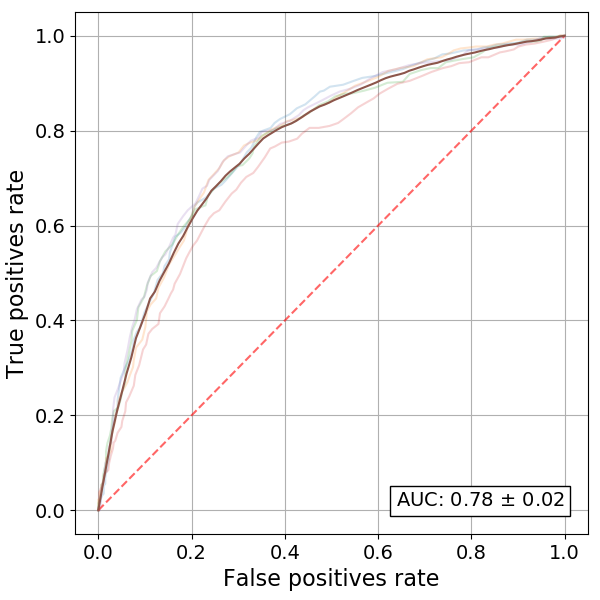 | | |
| --- | --- | --- | --- | --- | --- |
|  | Figure A5.1 – Log. R. ROC for file with series replaced with last values, missing data filtered out |  |  | Figure A5.2 – Log. R. ROC for file with series replaced with last values, missing data filled in |  |
| 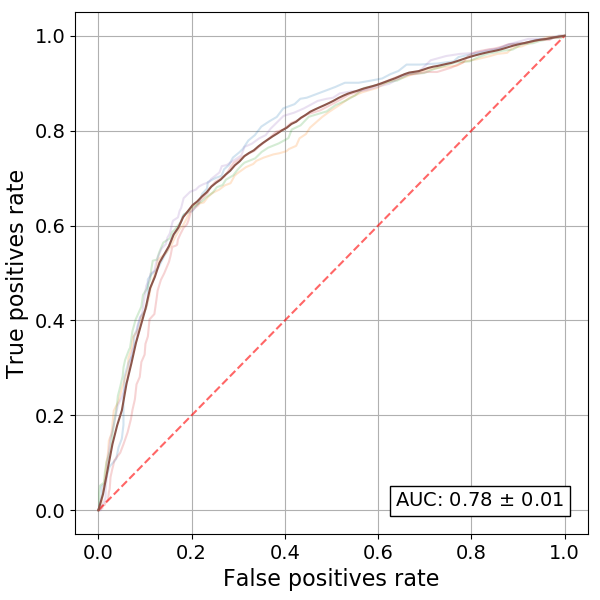 | | | 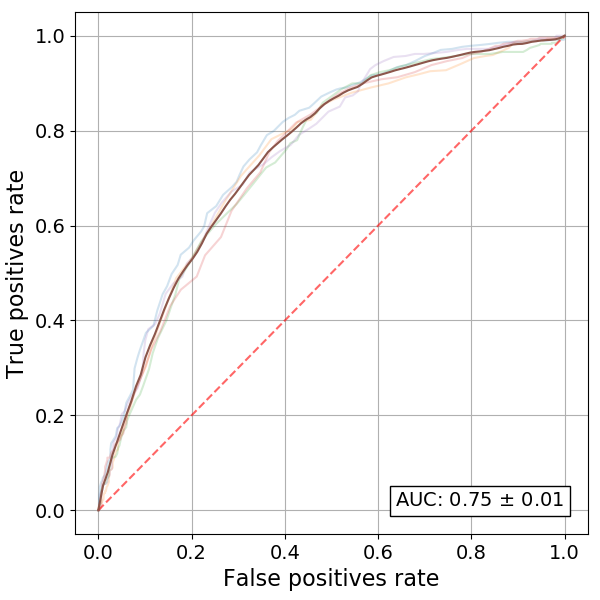 | | |
|  | Figure A5.3 – Log. R. ROC for file with series replaced with the stats, missing data filtered out |  |  | Figure A5.4 – Log. R. ROC for file with series replaced with the stats, missing data filled in |  |
| 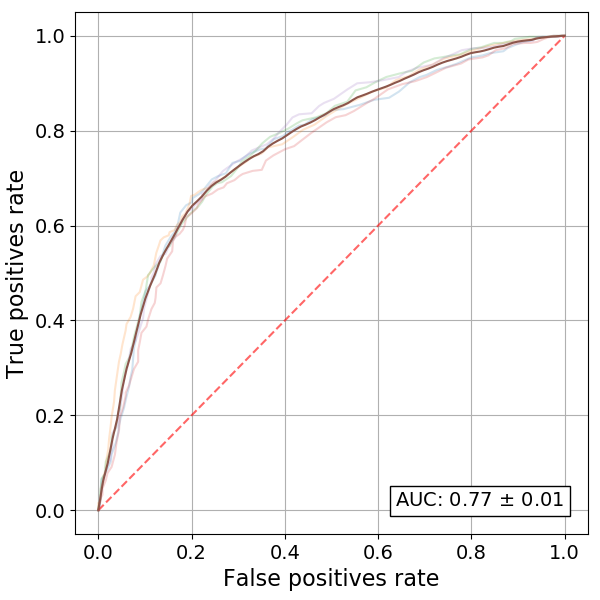 | | | 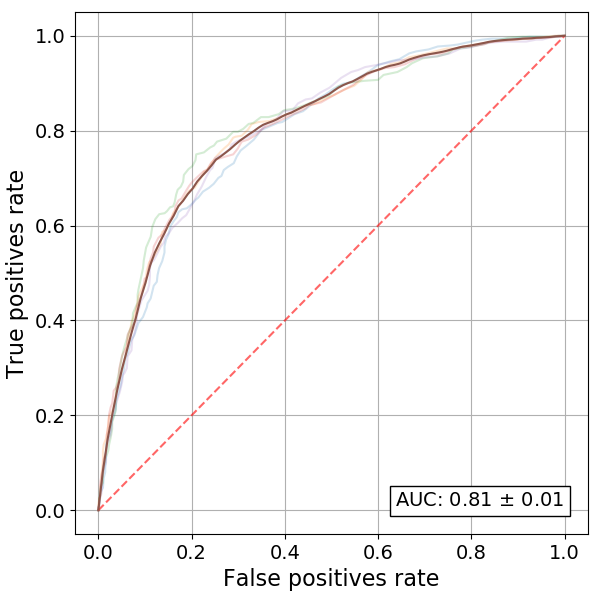 | | |
|  | Figure A5.5 – Log. R. ROC for file with series replaced with maximums, missing data filtered out |  |  | Figure A5.6 – Log. R. ROC for file with series replaced with maximums, missing data filled in |  |
